# Supplementary material for: Health-related quality of life and economic burden of childhood pneumonia in China: a multiregion study
Source: BMJ Paediatr Open. 2023 Nov 1;7(1):e002031. doi: 10.1136/bmjpo-2023-002031 (PMC10626790; doi:10.1136/bmjpo-2023-002031)
Supplement: Supplementary data [file bmjpo-2023-002031supp001.pdf]

Guardians' responses to the EQ-5D-Y dimensions at admission and discharge

Dimension	Overall		Shanghai		Zhengzhou		Kunming		P-value[a]		
	Admission N=394	Discharge N=372	Admission N=66	Discharge N=44	Admission N=120	Discharge N=120	Admission N=208	Discharge N=208	Admission	Discharge	Comparison[b]
**Walking about**									<0.01	0.06	<0.01
No problems	152 (38.6%)	202 (54.3%)	59 (89.4%)	44 (100.0%)	27 (22.5%)	66 (55.0%)	66 (31.7%)	92 (44.2%)			
A little bit of problems	86 (21.8%)	32 (8.6%)	5 (7.6%)	0	56 (46.7%)	27 (22.5%)	25 (12.0%)	5 (2.4%)			
Some problems	16 (4.1%)	6 (1.6%)	0	0	10 (8.3%)	6 (5.0%)	6 (2.9%)	0			
A lot of problems	7 (1.8%)	2 (0.5%)	0	0	6 (5.0%)	1 (0.8%)	1 (0.5%)	1 (0.5%)			
Extreme problems	1 (0.3%)	0	0	0	1 (0.8%)	0	0	0			
＜18month[c]	132（33.5%）	130（34.9%）	2 (3.0%)	0	20 (16.7%)	20 (16.7%)	110 (52.9%)	110 (52.9%)			
**Doing usual activities**									<0.01	<0.01	<0.01
No problems	146 (37.1%)	319 (85.8%)	36 (54.5%)	43 (97.7%)	20 (16.7%)	77 (64.2)	90 (43.3%)	199 (95.7%)			
A little bit of problems	185 (47.0%)	45 (12.1%)	30 (45.5%)	1 (2.3%)	67 (55.8%)	35 (29.2)	88 (42.3%)	9 (4.3%)			
Some problems	46 (11.7%)	7 (1.9%)	0	0	23 (19.2%)	7 (5.8)	23 (11.1%)	0			
A lot of problems	11 (2.8%)	1 (0.3%)	0	0	7 (5.8%)	1 (0.8)	4 (1.9%)	0			
Extreme problems	6 (1.5%)	0	0	0	3 (2.5%)	0	3 (1.4%)	0			
**Having pain or discomfort**									<0.01	<0.01	<0.01
No problems	56 (14.2%)	318 (85.5%)	6 (9.1%)	40 (90.9%)	4 (3.3%)	79 (65.8%)	46 (22.1%)	199 (95.7%)			
A little bit of problems	223 (56.6%)	43 (11.6%)	54 (81.8%)	4 (9.1%)	60 (50.0%)	30 (25.0%)	109 (52.4%)	9 (4.3%)			
Some problems	93 (23.6%)	9 (2.4%)	6 (9.1%)	0	42 (35.0%)	9 (7.5%)	45 (21.6%)	0			
A lot of problems	20 (5.1%)	2 (0.5%)	0	0	12 (10.0%)	2 (1.7%)	8 (3.8%)	0			
Extreme problems	2 (0.5%)	0	0	0	2 (1.7%)	0	0	0			
**Feeling worried, sad or unhappy**									<0.01	<0.01	<0.01
No problems	67 (17.0%)	301 (81.0%)	8 (12.1%)	22 (50.0%)	1 (0.8%)	78 (65.0%)	58 (27.9%)	201 (96.6%)			
A little bit of problems	193 (49.0%)	63 (16.9%)	33 (50.0%)	22 (50.0%)	58 (48.3%)	34 (28.3%)	102 (49.0%)	7 (3.4%)			
Some problems	101 (25.6%)	6 (1.6%)	25 (37.9%)	0	37 (30.8%)	6 (5.0%)	39 (18.8%)	0			
A lot of problems	33 (8.4%)	2 (0.5%)	0	0	24 (20.0%)	2 (1.7%)	9 (4.3%)	0			
Extreme problems	0	0	0	0	0	0	0	0			

[a]Calculated using the chi-square test.

[b]The comparison of the responses of the guardians on EQ-5D-Y dimensions at admission and discharge.

[c]Children younger than 18 months were assumed to have no problem on walking about dimension.
