# Supplementary material for: Health-related quality of life and economic burden of childhood pneumonia in China: a multiregion study
Source: BMJ Paediatr Open. 2023 Nov 1;7(1):e002031. doi: 10.1136/bmjpo-2023-002031 (PMC10626790; doi:10.1136/bmjpo-2023-002031)
Supplement: Author's manuscript [file bmjpo-2023-002031.draft_revisions.pdf]

BMJ Paediatrics Open

BMJ Paediatrics Open is committed to open peer review. As part of this commitment we make the peer review history of every article we publish publicly available.

When an article is published we post the peer reviewers' comments and the authors' responses online. We also post the versions of the paper that were used during peer review. These are the versions that the peer review comments apply to.

The versions of the paper that follow are the versions that were submitted during the peer review process. They are not the versions of record or the final published versions. They should not be cited or distributed as the published version of this manuscript.

BMJ Paediatrics Open is an open access journal and the full, final, typeset and author-corrected version of record of the manuscript is available on our site with no access controls, subscription charges or pay-per-view fees (http://bmjpaedsopen.bmj.com).

If you have any questions on BMJ Paediatrics Open's open peer review process please email info.bmjpo@bmj.com

BMJ Paediatrics Open

Health-related quality of life and economic burden of childhood pneumonia in China: a multi-region study

Journal:	*BMJ Paediatrics Open*
Manuscript ID	bmjpo-2023-002031
Article Type:	Original research
Date Submitted by the Author:	20-Apr-2023
Complete List of Authors:	Gao, Junyang; Fudan University<br>Fan, Jingzhi; Fudan University<br>Zhou, Huijun; University of Shanghai for Science and Technology<br>Jit, Mark; London School of Hygiene & Tropical Medicine<br>Wang, Pei; Fudan University
Keywords:	Health services research, Adolescent Health

SCHOLARONE™
Manuscripts

I, the Submitting Author has the right to grant and does grant on behalf of all authors of the Work (as defined in the below author licence), an exclusive licence and/or a non-exclusive licence for contributions from authors who are: i) UK Crown employees; ii) where BMJ has agreed a CC-BY licence shall apply, and/or iii) in accordance with the terms applicable for US Federal Government officers or employees acting as part of their official duties; on a worldwide, perpetual, irrevocable, royalty-free basis to BMJ Publishing Group Ltd ("BMJ") its licensees and where the relevant Journal is co-owned by BMJ to the co-owners of the Journal, to publish the Work in this journal and any other BMJ products and to exploit all rights, as set out in our licence.

The Submitting Author accepts and understands that any supply made under these terms is made by BMJ to the Submitting Author unless you are acting as an employee on behalf of your employer or a postgraduate student of an affiliated institution which is paying any applicable article publishing charge ("APC") for Open Access articles. Where the Submitting Author wishes to make the Work available on an Open Access basis (and intends to pay the relevant APC), the terms of reuse of such Open Access shall be governed by a Creative Commons licence – details of these licences and which Creative Commons licence will apply to this Work are set out in our licence referred to above.

Other than as permitted in any relevant BMJ Author's Self Archiving Policies, I confirm this Work has not been accepted for publication elsewhere, is not being considered for publication elsewhere and does not duplicate material already published. I confirm all authors consent to publication of this Work and authorise the granting of this licence.

Confidential: For Review Only

Health-related quality of life and economic burden of childhood pneumonia in China: a multi-region study

Junyang Gao [1,2*], BSc

Jingzhi Fan [1,2*], BSc

Huijun Zhou[3], PhD

Mark Jit[4], PhD

Pei Wang[1,2], PhD

[1] School of Public Health, Fudan University, Shanghai 200032, China

[2] Key Lab of Health Technology Assessment, National Health Commission of the People's Republic of China (Fudan University), Shanghai, China

[3] Business School, University of Shanghai for Science and Technology, Shanghai, China

4Department of Infectious Disease Epidemiology, Faculty of Epidemiology and Population Health, London School of Hygiene & Tropical Medicine, London, United Kingdom.

[*] The authors contribute equally

Corresponding to:

Pei Wang,

School of Public Health,

Fudan University

130 Dong An Road, Shanghai 200032, China.

E-mail: wang_p@fudan.edu.cn.

Text word count: 3122 words

Keywords: pneumonia, child, health-related quality of life, economic burden

Confidential: For Review Only

Abstract

Objective: To evaluate the health utility and total cost associated with hospitalization for treating pediatric pneumonia in China; and to explore the associated factors.

Methods: The study recruited a series of children under 5 years hospitalized for pneumonia in Shanghai, Zhengzhou, and Kunming from January to October 2019.

Health utility was assessed using the proxy version of EQ-5D-Y by interviewing patients' guardians face-to-face. The assessment was administered twice at patients' admission and discharge. Cost incurred for receiving the hospitalization were collected. Multiple linear regression was used to explore factors of EQ-5D-Y health utility score (HUS) and costs.

Results: A total of 501 pediatric patients with a mean age (SD) of 1.85 (1.34) years were included in the analysis. The mean HUS (SD) of the patients was 0.78 (0.18) at admission, and increased to 0.96 (0.10) at discharge. Some patients (14.2%) still felt worried, sad or unhappy after hospitalization. The mean hospitalization cost and total cost were RMB 5,859 and RMB 6,439, respectively. The HUS was lower and the economic burden was heavier for the children in Zhengzhou. Apart from region, type of work and education level of guardians were also related to the baseline HUS and HUS increment after treatment, respectively; insurance status, guardians' employment and hospitalization days were associated with the costs.

Conclusion: The children with pneumonia have poor baseline HRQOL, and many of them still have mental health problems after treatment. The economic burden varied significantly across regions and is heavy for the patients' families in less developed areas (i.e., Zhengzhou and Kunming).

Keywords: pneumonia, child, health-related quality of life, economic burden

Confidential: For Review Only

Introduction

Pneumonia is a significant type of lower respiratory tract infection that is caused by different pathogens such as bacteria and viruses(1). The disease burden is more serious in developing countries. In 2019, the number of pneumonia and severe pneumonia cases in five Asian and African countries (i.e., India, Nigeria, Indonesia, Pakistan and China) accounted for 54% of the total cases globally(2); studies have also shown that pneumonia is one of the top 5 causes of death in children under 5 years in China(3, 4). Pneumonia causes not only physical impairment, but also psychological, debilitating and social adjustment problems to the affected child. Hence, health-related quality of life (HRQOL), a comprehensive health outcome measure is required to holistically reflect the disease influence. Moreover, the HRQOL information could also be translated into health utility score (HUS) reflecting the value of HRQOL for use in economic evaluation if it is measured by utility instruments such as EQ-5D.

International and domestic studies have assessed HRQOL of pediatric patients with pneumonia using HRQOL instruments such as the Children's Quality of Life Scale (PQL 4.0)(5, 6), the Generic Quality of Life Inventory-74 (GQOLI-74)(7). They found that the pediatric patients had poorer HRQOL, especially in social and psychological functioning dimensions. Studies in Indonesia and Thailand have also reported HUS of the patients using the proxy version of EQ-5D(8, 9); while no studies have yet provided HUS for the Chinese patients.

In addition to its HRQOL impact, pneumonia also places a heavy financial burden on the children's family. Several studies have assessed its economic burden in childhood respiratory infections and pneumonia in China(10, 11). Wang et al. investigated the average length of stay (LOS) in hospital and hospitalization cost of 8,334 children with acute respiratory disease under 5 years in Gansu province from 2015 to 2018. They found that the LOS was 6.6 days and the expenditure was RMB 5,613 exceeding 30% of the per capita disposable income of the province in 2018. Similarly, Wang et al. conducted a retrospective study of 86 children with pneumonia under 5 years in a community in Shanghai during 2012. The study indicated the average total cost including outpatient cost, hospitalization cost, out-of-pocket drug cost, traffic cost, lost work cost was RMB 4,017, which accounted for 10% of the per capita disposable income of Shanghai in

https://mc.manuscriptcentral.com/bmjpo

Confidential: For Review Only

2012. However, the studies were based on the patients in a single place, which may be under-represented and lead to limited extrapolation.

Hence, this study aimed to systematically investigate the HRQOL and economic burden of the children with pneumonia from three regions with divergent economic development levels in China.

Methods

Study design and patients

This study measured HRQOL of hospitalized pneumonia children at admission and discharge. Various costs associated with hospitalization were collected and aggregated. The study was conducted from January to October 2019 in three provincial capitals of sampled provinces representing different socio-economics statuses. The cities were Shanghai (eastern region), Zhengzhou (central region), and Kunming (western region), representing high, medium, and low status, respectively. In each city, a general or special (e.g., children's hospital) tertiary hospital was selected as the sampling hospital.

The inclusion criteria for the study were 1) younger than five years old, 2) clinical diagnosis of pneumonia, and 3) no concomitant diseases. All the pediatric patients hospitalized in the studying sites at the time of study were assessed for the eligibility by trained interviewers. Once a patient was eligible, his/her parents/guardians were invited to participate in the study.

The consenting parents/guardians were interviewed face-to-face twice. The first interview was conducted within the first two days of patients' admission. A questionnaire was used to assess the child's HRQOL and to collect information on the child's (i.e., gender, age and medical insurance) and his/her guardian's sociodemographic characteristics (i.e., gender, age, education level, marriage status, employment, and monthly income level, race, domicile, religion, relationship with the child). The second interview was performed when the child was discharged from the hospital, and assessed the child's HRQOL again, as well as the total hospitalization costs including any household expenses, insurance payments (if any), lost work time, and the length of stay (LOS).

https://mc.manuscriptcentral.com/bmjpo

Confidential: For Review Only

The study was supported by grants from the National Institute for Health Research (NIHR) (grant no. 16/137/109) using UK aid from the UK Government to support global health research. The views expressed in this publication are those of the author(s) and not necessarily those of the NIHR or the UK Department of Health and Social Care.

Health-related quality-of-life Measurement

The EQ-5D-Y is used as the HRQOL measure in the study. It is the youth version of the widely used EQ-5D that is more aligned with children's perceptions and understanding. It has five dimensions each with five response options: *walking about*, *looking after myself*, *doing usual activity*, *having pain or discomfort*, *feeling worried*, *sad or unhappy*.

The five response options of Y-5L are *no problems* (level 1), *a little bit of problems* (level 2), *some problems* (level 3), *a lot of problems* (level 4), *extreme problem*s (level 5)(12). As a result, it defines a total of 3,125 (5^5) health states by combing the responses of each dimension. A HUS can be assigned to each health state using a utility value set. Since the utility value set for the Y-5L is not available currently, the study used the Chinese value set of the EQ-5D-5L to calculate the HUS for the Y-5L health states(13). Y-5L designs a visual analog scale (VAS) with a score of 100 at the top for "best imaginable health" and 0 at the bottom for "worst imaginable health".

Given that the children aged 0-5 years were cared by their parents, it was assumed that the dimension “looking after myself” is irrelevant. For the children under 18 months who were unable to stand, the dimension “walking about” is assumed irrelevant(14).

Economic Burden of Pneumonia

The economic burden of pneumonia was estimated by calculating direct medial cost (i.e., hospitalization cost including out-of-pocket cost, insurance payments if covered by insurance), non-medical cost (i.e., traffic cost), as well as indirect cost. Indirect cost was derived by estimating the loss of productivity of guardian using human capital approach. That is, the loss of work hour multiplies hourly wages based on annual average income in China in 2021 (8 hour per work day, and 250 work days per year). All cost data was adjusted to 2021 Chinese CNY using the latest published CPI index in China.

https://mc.manuscriptcentral.com/bmjpo

Confidential: For Review Only

Statistical analysis

Descriptive statistical analyses were adopted to depict sociodemographic characteristics of the patients and their guardians, distributions of Y-5L dimension responses, Y-5L HUS and VAS score, as well as economic burden data. Categorical variables were presented using number and percentages, and continuous variables were described using mean, standard deviations (SD) and range. Chi-square test or ANOVA test were used to compare the characteristics of the patients/guardians, HRQOL and economic burden of the patient among the three cities, and HRQOL between admission and discharge whenever appropriate. Comparison of HUS, VAS score and cost using the Tukey post hoc test(15) between samples were further conducted to determine which pairwise groups were different.

Multiple linear regression was used to analyze the factors influencing the HUS and economic burden of children with pneumonia. For the former, HUS at baseline and its difference between baseline and discharge were used as the two dependent variables. Demographic characteristics including region, gender, age, domicile, insurance status of the children; and religion, education level, marriage status, employment, monthly income level of the guardians were adopted as the independent variables for baseline HUS. For HUS difference, the independent variables included the variables for baseline HUS plus time loss from work of the guardians and hospital days of the children. For the economic burden, hospitalization cost and total cost (hospitalization cost, transportation cost, and cost for productivity loss) were used as the two dependent variables. The independent variables included in the two models were hospital days as well as the demographic characteristics mentioned above. Due to the non-normal distribution of the data, a natural logarithmic transformation of the costs was performed.

SPSS 23.0 was used for all the analysis, and the threshold for significant differences was a P-value less than 0.05.

Confidential: For Review Only

Results

Characteristics of study subjects

Table 1 shows the sociodemographic characteristics of children with pneumonia and their guardians. A total of 501 patients were included in analysis: 173, 120 and 208 in Shanghai, Zhengzhou and Kunming, respectively. The mean (SD) age of the patients was 1.85 (1.34) years, and the proportions of boys and girls were close (51.5% vs. 48.5%). A relatively high percentage of the children were without medical insurance (48.5%). More than half of the guardians were female (53.9%) with a mean (SD) age of 33.49 (9.06) years, and the majority of them were parents of the children (91.6%). They were mostly Han Chinese (90.6%), non-religious (96.8%), married (98.0%), from urban area (67.5%), university-educated or above (42.5%), enterprise/self-employed (41.9%). Most of them (57.8%) had family monthly income over 10,000 CNY. The characteristics of the patients and their guardians differed among the three regions except for marital status (P=0.25).

Health-related quality-of-life of the children

The percentage of reporting problems in the Y-5L dimensions at admission and discharge is shown in Table 2. At admission, the children had more problems in *having pain or discomfort* (67.5%) and *feeling sad, worried, or unhappy* (65.3%) dimensions; and the children in Zhengzhou had the highest prevalence of problems in each dimension. At discharge, the children's health status was significantly improved and no cases had very severe problems (level 5) in all the dimensions, while a relatively high proportion of problems was still observed in *feeling worried, sad or unhappy* dimension (14.2%). Similarly, the children in Zhengzhou tended to have more problems.

Table 3 reports the Y-5L HUS and VAS score of children with pneumonia in the three locations. The mean HUS of the patients at admission was 0.78, and the patients in Shanghai and Zhengzhou reported the highest (0.84) and lowest HUS (0.66), respectively. The HUS at discharge was significantly increased with the mean value being 0.96, and the children from Kunming and Zhengzhou had higher (0.99) or lower (0.90) HUS. The results of multiple comparisons showed

Confidential: For Review Only

significant difference in HUS between Shanghai and Zhengzhou, Kunming and Zhengzhou at both admission and discharge, while the difference between Shanghai and Kunming was insignificant. The difference in HUS at admission and discharge for children in Zhengzhou was significantly greater than the difference in the other two regions. The mean VAS score of children in the three sites at both admission and discharge was also significantly different (62.9 vs. 89.2, P<0.05), with the children in Kunming having the highest score at both admission (65.4) and discharge (91.9), those in Zhengzhou and Shanghai having the lowest score at admission (58.3) and at discharge (77.2), respectively

Economic Burden of Pneumonia

The economic burden manifested by hospitalization cost and total cost summing of hospitalization cost, transportation cost, and indirect cost are shown in Table 4. The mean hospitalization cost of the three regions was RMB 5,859 and the children in Zhengzhou (RMB 8,667) had much higher cost than that in Shanghai (RMB 3,100) and Kunming (RMB 6,522). The mean transportation cost was RMB 110, and the children in Kunming (RMB 220) had the highest cost followed by the children in Shanghai (RMB 4) and Zhengzhou (RMB 73). The average hospitalization days was 8.0 days: the days were 10.6 days, 8.2 days, and 4.8 days for the children in Kunming, Shanghai, and Zhengzhou, respectively. Correspondingly, the average time of loss work of guardians in Kunming (65.9 hours) was substantially higher than that in Shanghai (6.5 hours) and Zhengzhou (33.1 hours). This also resulted in the highest indirect cost in Kunming (RMB 710), followed by Zhengzhou (RMB 444), and Shanghai (RMB 187). For the total cost, the average amount was RMB 6,439, and the patients in Zhengzhou had the highest amount (RMB 9,183), followed by Kunming (RMB 7,464), and Shanghai (RMB 3,293).

Regression analyses

Table 5 and Table 6 present the significant coefficients generated from the multiple linear regressions. Region and type of work were significantly associated with baseline HUS (Table 5). That is, the children in Zhengzhou, and their guardians who were temporary workers had lower baseline HUS. Region and education level of guardians were significantly correlated with the difference in HUS: the children in Zhengzhou and with guardians who received university and

https://mc.manuscriptcentral.com/bmjpo

Confidential: For Review Only

above education were more likely to have higher or lower difference in HUS, respectively (Table 5).

For economic burden, region, insurance status, employment and hospital days were significantly associated with both hospitalization and total costs (Table 6). Specifically, the children in Zhengzhou and Kunming had higher costs. The two costs were significantly lower for the children without insurance and whose guardians were civil servant, public institution worker or temporary work, respectively, and hospitalization cost was also lower for the children with farming guardians. The children with longer hospitalization days also had higher costs.

Discussion

To our knowledge, this is the first study comprehensively assessing HRQOL and economic burden of the Chinese children with pneumonia aged 0-5 years. We found that the children with pneumonia had inferior health status both physically and psychologically; and the disease economic burden varied greatly among regions. Moreover, we also identified several important factors influencing the HUS and economic burden. Therefore, this study deepens the understanding of HRQOL and economic burden of the children in China.

At admission, the majority of children had health problems especially in *having pain or discomfort*, *feeling worried*, *sad or unhappy* dimensions as well as low HUS and VAS score. Previous studies have also reported similar findings that the pediatric patients with pneumonia had poor HRQOL at admission, especially in social and psychological functioning dimensions(5, 6). When pneumonia occurred, the children had symptoms such as fever, cough, sore throat and breathlessness(16), which may bring physical discomfort to the children, affect their emotions and lead to psychological problems. We further found that although their health improved a lot at discharge, many of them were still feeling worried, sad, or unhappy at discharge. Thus, their psychological condition should deserve more attention in the post-discharge period.

Among the three regions, the children in Zhengzhou had worse baseline HUS, which was probably due to that they were mainly from urban area containing more risk factors that may contribute to the development of pneumonia in childhood(17). The children also had poorer

Confidential: For Review Only

baseline HUS when their guardians were temporary workers. An Israeli study showed that both parental unemployment and workday loss affected the HRQOL of children with pneumonia as well(18). Temporary work means unstable income, making it difficult to provide good care to the children. The children in Zhengzhou also had larger HUS difference; while the HUS difference of children whose guardians' education was university and above was smaller. Since the patients were discharged from hospitals because their health was recovered and their HUS at discharge was close to full health, the identified association could be attributed to the HUS at baseline. That is, the children in Zhengzhou had worse baseline HUS; and the children with highly educated guardians may be better cared thus with better baseline health.

We found that the average hospitalization and total costs were comparable to the previous results for the Chinese children under 5 years with pneumonia(10, 11): the average hospitalization cost was RMB 5,771 in Gansu and the average total cost was RMB 4,642 in Shanghai after being adjusted for 2021 CNY. The costs were also similar to the costs of other common childhood infectious diseases in China. According to 2021 CNY, the average hospitalization costs for children in Shenyang with scarlet fever and hand, foot and mouth disease were RMB 4,979 and RMB 5,050, respectively(19); the average total cost for inpatient cases with chickenpox in school-age children was RMB 3,312 in Shenzhen(20). On the other hand, we identified the disease economic burden varied greatly among the regions: the total cost was calculated to be 1.9% of the local per capita GDP in Shanghai, 9.6% in Zhengzhou, and 8.7% in Kunming, respectively. The finding indicated that the disease places severer economic burden on the children's families in less economically developed areas. The government thus could take necessary activites (e.g., increasing the reimbursement rate of medical insurance) to alleviate the burden of the families.

The children in Zhengzhou had higher hospitalization cost. This may be due to their worse baseline health status, requiring more medical resources and thus higher costs. For the transportation and indirect costs, the patients in Kunming had higher amount. Kunming is the capital city of Yunnan province, which had a relatively low level of socio-economic development and a lack of medical resources compared to other regions of China. Hence, the patients in other cities of the province have to concentrate in Kunming, leading to the higher costs.

Confidential: For Review Only

The children without medical insurance had significantly lower hospitalization and total costs. A previous study had indicated that relying solely on out-of-pocket payment provided obstacles for treatment access, skewing treatment seeking towards those affordable(21), leading to lower costs. The two costs were also significantly associated with hospitalization days, and similar findings had been reported in a previous study that shortening the number of ineffective hospitalization days helped to reduce the financial burden(22). In the study, we found that hospitalization and total costs were significantly higher for the children whose guardians were enterprise or self-employed workers. This was probably because they may have better economic conditions and were willing to spend more for their children to get better treatment.

This study has several limitations which should be noted. First, the Chinese Y-5L value set has not been available, so the Chinese EQ-5D-5L value set was used to calculate the HUS instead. In addition, the Y-5L was designed mainly for children over 6 years of age, and the reliability and validity of its proxy version need to be further tested when it is applied to the guadians of children under 5 years. Second, although we excluded the pneumonia patients with other acute diseases, there may be undiagnosed comorbidities during data collection, which may affect HRQOL scores. Third, we did not collect certain clinical characteristics including disease severity and treatment modality, thus their influence on HRQOL and economic burden cannot be assessed.

Conclusion

The pediatric patients with pneumonia mainly suffered from pain, discomfort and felt sad, worried, or unhappy at admission, and many of them still had mental health problems after treatment. Region and education level of guardians were the two factors influencing their HRQOL. The economic burden of the disease varied significantly across regions and was heavy for the patients' families in less developed areas. Insurance status of the patients, employment status of their guardians, and hospitalization days were also associated with the burden.

Confidential: For Review Only

Acknowledgements: We gratefully acknowledge the study participants and their families. We thank the hospitals for their support and cooperation with our data collection.

Contributors: JYG: Methodology, data analysis, manuscript writing (original draft). JZF: Data analysis, manuscript writing (original draft and revision). PW: Conceptualization, methodology, critical manuscript revision, supervision and project administration. All authors critically revised the manuscript and approved the final version of manuscript.

Funding: This research was funded by the National Institute for Health Research (NIHR) (GHR 16/137/09) using UK aid from the UK Government to support global health research. The views expressed in this publication are those of the author(s) and not necessarily those of the NIHR or the UK government.

Ethic: This study was conducted according to the guidelines of the Declaration of Helsinki and approved by the Institutional Review Board of School of Public Health, Fudan University (IRB00002408).

Competing interests: The authors declare no competing interests

Patient and public involvement: Patients and/or the public were not involved in the design, conduct, reporting or dissemination plans of this research

Provenance and peer review: Not commissioned; externally peer reviewed.

Data availability: The datasets analyzed during the current study are available from the corresponding author on reasonable request

Confidential: For Review Only

Table 1 Sociodemographic characteristics of children with pneumonia and their guardians among the three regions

Variables	Overall N=501	Shanghai N=173	Zhengzhou N=120	Kunming N=208	P-value[a]
**Characteristics of the child**					
**Gender**					< 0.01
Boys	258 (51.5)	72 (41.6)	63 (52.5)	123 (59.1)	
Girls	243 (48.5)	101 (58.4)	57 (47.5)	85 (40.9)	
**Age(year)**					< 0.01
Mean (SD)	1.85 (1.34)	1.67 (1.20)	2.70 (1.19)	1.51 (1.33)	
Range	0.03-5.00	0.42-5.00	0.50-5.00	0.03-5.00	
**Medical Insurance**					< 0.01
Yes	185 (36.9)	91 (52.6)	41 (34.2)	53 (25.5)	
None	243 (48.5)	80 (46.2)	79 (65.8)	84 (40.4)	
Unknown	71 (14.2)	-	-	71 (34.1)	
**Guardianship characteristics**					
**Age(year)**					0.01
Mean (SD)	33.49 (9.06)	36.28 (12.63)	32.52 (3.63)	31.71 (6.93)	
Range	14-69	22-69	25-41	14-59	
**Gender**					< 0.01
Male	209 (41.7)	57 (32.9)	42 (35.0)	110 (52.9)	
Female	292 (58.3)	116 (67.1)	78 (65.0)	98 (47.1)	
**Relationship**					< 0.01
Father	189 (37.7)	35 (20.2)	42 (35.0)	112 (53.8)	
Mother	270 (53.9)	105 (60.7)	78 (65.0)	87 (41.8)	
Other[b]	42 (8.4)	33 (19.1)	-	9 (4.3)	
**Ethnic**					< 0.01
Han	454 (90.6)	171 (98.8)	118 (98.3)	165 (79.3)	
Others	47 (9.4)	2 (1.2)	2 (1.7)	43 (20.7)	
**Religion**					0.01
None	485 (96.8)	170 (98.3)	118 (98.3)	197 (94.7)	
Yes or refused to answer	13 (2.6)	3 (1.7)	2 (1.6)	11 (5.3)	
**Marriage Status**					0.25
Married	491 (98.0)	170 (98.3)	120 (100.0)	201 (96.6)	
Singles and others	10 (2.0)	3 (1.8)	-	7 (3.4)	
**Domicile**					< 0.01
City	241 (48.1)	102 (59.0)	95 (79.2)	44 (21.2)	
Town	97 (19.4)	43 (24.9)	20 (16.7)	34 (16.3)	
Rural	161 (32.1)	27 (15.6)	5 (4.2)	129 (62.0)	
**Education**					< 0.01
Elementary school and below	28 (5.6)	11 (6.3)	-	17 (8.2)	
Junior High School	104 (20.8)	14 (8.1)	2 (1.7)	88 (42.3)	
High School	88 (17.6)	30 (17.3)	15 (12.5)	43 (20.7)	

Confidential: For Review Only

College	68 (13.6)	23 (13.3)	25 (20.8)	20 (9.6)	
University and above	213 (42.5)	95 (54.9)	78 (75.0)	40 (19.2)	
**Employment**					< 0.01
Enterprise/self-employed	210 (41.9)	77 (44.5)	83 (69.2)	50 (24.0)	
Civil servants / Public Institution	83 (16.6)	20 (11.6)	24 (20.0)	39 (18.8)	
Full-time home/housewife	73 (14.6)	37 (21.4)	5 (4.2)	31 (14.9)	
Retirement	21 (4.2)	18 (10.4)	-	3 (1.4)	
Farming	50 (10.0)	11 (6.4)	3 (2.5)	36 (17.3)	
Temporary workers	39 (7.8)	1 (0.6)	4 (3.3)	34 (16.3)	
Unemployment	21 (4.2)	7 (4.1)	1 (0.8)	13 (6.2)	
Other or refused to answer	3 (0.6)	1 (0.6)	-	2 (1.0)	
**Family monthly income (CNY)**					< 0.01
<5000	83 (16.6)	-	3 (2.5)	80 (38.5)	
5000-10000	97 (19.4)	-	20 (16.7)	77 (37.0)	
10000-20000	76 (15.2)	9 (5.2)	47 (39.2)	20 (9.6)	
20,000-30,000	75 (15.0)	37 (21.4)	34 (28.3)	4 (1.9)	
> 30,000	117 (23.4)	94 (54.3)	16 (13.3)	7 (3.3)	
Unknown or refused to answer	52 (10.4)	32 (18.5)	-	20 (9.6)	

SD: standard deviations; CNY: Chinese Yuan.

[a]Comparison of population characteristics among the three regions. Categorical variables and continuous variables were analyzed using chi-square test and ANOVA test, respectively.

[b]This refers to a guardian such as a non-parental relative of the affected child.

Confidential: For Review Only

Table 2 Guardians' responses to the EQ-5D-Y dimensions at admission and discharge.

Dimension	Overall (N=501)		Shanghai (N=173)		Zhengzhou (N=120)		Kunming (N=208)		P-value[a]		
	Admission	Discharge	Admission	Discharge	Admission	Discharge	Admission	Discharge	Admission	Discharge	Comparison[b]
**Walking about[c]**									<0.01	0.06	<0.01
No problems	262 (52.3%)	312 (62.3%)	59 (34.1%)	44 (25.4%)	27 (22.5%)	66 (55.0%)	176 (84.6%)	202 (97.1%)			
A little bit of problems	86 (17.2%)	32 (6.4%)	5 (2.9%)	-	56 (46.7%)	27 (22.5%)	25 (12.0%)	5 (2.4%)			
Some problems	16 (3.2%)	6 (1.2%)	-	-	10 (8.3%)	6 (5.0%)	6 (2.9%)	-			
A lot of problems	7 (1.4%)	2 (0.4%)	-	-	6 (5.0%)	1 (0.8%)	1 (0.5%)	1 (0.5%)			
Extreme problems	1 (0.2%)	-	-	-	1 (0.8%)	-	-	-			
**Doing usual activities**									<0.01	<0.01	<0.01
No problems	146 (29.1%)	319 (63.7%)	36 (20.8%)	43 (24.9%)	20 (16.7%)	77 (64.2)	90 (43.3%)	199 (95.7%)			
A little bit of problems	185 (36.9%)	45 (9.0%)	30 (17.3%)	1 (0.6%)	67 (55.8%)	35 (29.2)	88 (42.3%)	9 (4.3%)			
Some problems	46 (9.2%)	7 (1.4%)	-	-	23 (19.2%)	7 (5.8)	23 (11.1%)	-			
A lot of problems	11 (2.2%)	1 (0.2%)	-	-	7 (5.8%)	1 (0.8)	4 (1.9%)	-			
Extreme problems	6 (1.2%)	-	-	-	3 (2.5%)	-	3 (1.4%)	-			
**Having pain or discomfort**									<0.01	<0.01	<0.01
No problems	56 (11.2%)	318 (63.5%)	6 (3.5%)	40 (23.1%)	4 (3.3%)	79 (65.8%)	46 (22.1%)	199 (95.7%)			
A little bit of problems	223 (44.5%)	43 (8.6%)	54 (31.2%)	4 (2.3%)	60 (50.0%)	30 (25.0%)	109 (52.4%)	9 (4.3%)			
Some problems	93 (18.6%)	9 (1.8%)	6 (3.5%)	-	42 (35.0%)	9 (7.5%)	45 (21.6%)	-			
A lot of problems	20 (4.0%)	2 (0.4%)	-	-	12 (10.0%)	2 (1.7%)	8 (3.8%)	-			
Extreme problems	2 (0.4%)	-	-	-	2 (1.7%)	-	-	-			
**Feeling worried, sad or unhappy**									<0.01	<0.01	<0.01

Confidential: For Review Only

No problems	67 (13.4%)	301 (60.1%)	8 (4.6%)	22 (12.7%)	1 (0.8%)	78 (65.0%)	58 (27.9%)	201 (96.6%)
A little bit of problems	193 (38.5%)	63 (12.6%)	33 (19.1%)	22 (12.7%)	58 (48.3%)	34 (28.3%)	102 (49.0%)	7 (3.4%)
Some problems	101 (20.2%)	6 (1.2%)	25 (14.5%)	-	37 (30.8%)	6 (5.0%)	39 (18.8%)	-
A lot of problems	33 (6.6%)	2 (0.4%)	-	-	24 (20.0%)	2 (1.7%)	9 (4.3%)	-
Extreme problems		-	-	-	-	-	-	-

[a]Calculated using the chi-square test.

[b]The comparison of the responses of the guardians on EQ-5D-Y dimensions at admission and discharge.

[c]Children younger than 18 months were assumed to have no problem on walking about dimension.

Confidential: For Review Only

Table 3 EQ-5D-Y health utility and EQ-VAS scores of children with pneumonia in the three locations

	Total N=501	Shanghai (1) N=173	Zhengzhou (2) N=120	Kunming (3) N=208	Post hoc analysis[a]	P-value[b]
**Utility baseline**						
Mean (SD)	0.78 (0.18)	0.84 (0.07)	0.66 (0.21)	0.82 (0.15)	(1) > (2); (3) > (2)	<0.001
Range	-0.04-1.00	0.63-1.00	-0.04-0.95	0.25-1.00		
**VAS baseline**						
Mean (SD)	62. 9（16. 6）	63. 5（14. 2）	58. 3（15. 5）	65. 4（17. 4）	（3）＞（2）	＜0. 01
Range	15–100	40–95	15–84	20–100		
**Utility discharge**						
Mean (SD)	0. 96（0. 10）	0. 97（0. 04）	0. 90（0. 15）	0. 99（0. 04）	（1）＞（2）；（3）＞（2）	<0.001
Range	0. 27–1. 00	0. 85–1. 00	0. 27–1. 00	0. 67–1. 00		
**VAS discharge**						
Mean (SD)	89. 2（13. 0）	77. 2（9. 5）	88. 9（10. 7）	91. 9（13. 5）	（2）＞（1）；（3）＞（1）	＜0. 01
Range	40–100	60–95	47–100	40–100		
**Utility difference**						
Mean (SD)	0. 19（0. 16）	0. 15（0. 06）	0. 24（0. 21）	0. 17（0. 14）	（1）＜（2）；（2）＞（3）	<0.001
Range	–0. 05–1. 04	0. 04–0. 32	0–1. 04	–0. 05–0. 75		
**VAS difference**						
Mean (SD)	27. 1（13. 5）	20. 1（7. 7）	30. 6（14. 3）	26. 5（13. 4）	（2）＞（1）；（2）＞（3）	＜0. 01
Range	0–74	5–40	10–74	0–60		

SD, standard deviations.

[a]Comparison using the Tukey post hoc test.

[b]Calculated using ANOVA test.

Confidential: For Review Only

Table 4 Economic burden of disease in children with pneumonia

	Overall N=501	Shanghai (1)	Zhengzhou (2)	Kunming (3)	Post hoc comparison[a]	P value[b]
**Traffic cost**					(1) < (3); (2) < (3)	<0.01
Mean (SD)	110.0 (270.1)	4.3 (9.8)	72.9 (138.2)	219.9 (378.6)		
Range	0-3514	0-60	0-780	0-3514		
**Out-of-pocket cost**						
Mean (SD)	3816.1(3981.9)	2036.6 (1077.7)	3813.8(1990.3)	5229.1 (5473.3)	(1)<(2)<(3)	<0.01
Range	471-60420	471-8236	1350-10416	504-60420		
**Hospitalization cost**					(1) < (2); (1) < (3); (2) > (3)	
Mean (SD)	5859.2 (5486.5)	3099.7 (1474.2)	8666.6(3965.2)	6521.5 (7098.9)		<0.01
Range	302-80560	987-8261	3114-18998	302-80560		
**Loss of work time (hour)**						<0.01
Mean (SD)	37.9 (81.1)	6.5 (3.9)	33.1 (15.8)	65.9 (121.0)		
Range	0-1200	0-10	9-80	0-1200	-	
**Indirect cost[c]**						
Mean (SD)	465.79 (967.89)	187.40 (171.73)	443.50 (215.60)	710.20 (1444.48)	(1) < (3); (2) < (3)	<0.01
Range	0-15399.60	0-390.14	107.24-1072.44	0-15399.60		
**Hospitalization days**						<0.01
Mean (SD)	8.0 (6.6)	4.8 (1.9)	8.2 (3.0)	10.6(9.1)	-	
Range	0-50	1-11	5-15	0-50		
**Total Cost[d]**						<0.01
Mean (SD)	6439.37 (5772.99)	3292.52 (1511.23)	9183.00 (4085.72)	7463.64 (7437.87)	(1)<(3)<(2)	
Range	918.09-80560.48	986.86-8701.53	3370.06-19346.78	918.09-80560.48		

SD, standard deviations.

[a]Comparison using the Tukey post hoc test.

[b]Calculated using ANOVA test.

[c]Indirect cost: loss of work hour multiples income per hour that converted from annual average income in China 2021 (8 hour per work day, and 250 work days per year).

[d]Total cost includes hospitalization cost, traffic cost and indirect cost.

Confidential: For Review Only

Table 5 Multiple regression analysis of significant factors of baseline health utility score and health utility score difference between baseline and follow-up

Variables	Baseline HUS		HUS difference (discharge-admission)	
	**Coefficient (95% CI)[a]**	**P-value[a]**	**Coefficient (95% CI)[a]**	**P-value[a]**
**Region (Shanghai[b])**				
Zhengzhou	-0.17 (-0.23 , -0.11)	<0.01	0.08 (0.01 , 0.15)	0.03
Kunming	0.01 (-0.06 , 0.08)	0.69	-0.003 (-0.08 , 0.08)	0.94
**Education**				
**（Elementary school and below[b]）**				
Junior High School	-	-	-0.02 (-0.14,0.09)	0.69
High School	-	-	-0.004 (-0.08 , 0.07)	0.91
College	-	-	0.04 (-0.02 , 0.1)	0.15
University and above	-	-	-0.05 (-0.11 , 0)	0.05
**Employment Status**				
**(Enterprise/self-employed[b])**				
Civil servants / Public Institution	-0.03 (-0.08 , 0.01)	0.16	-	-
Full-time home/housewife	-0.03 (-0.1 , 0.04)	0.45	-	-
Retirement	-0.003 (-0.14 , 0.03)	0.96	-	-
Farming	-0.06 (-0.2 , -0.05)	0.21	-	-
Temporary workers	-0.12 (-0.15 , 0.05)	<0.01	-	-
Unemployment	-0.05 (-0.153 , 0.051)	0.33	-	-

Abbreviations: HUS, health utility score; CI, confidence interval.

[a]Calculated using multiple linear regression.

[b]Reference category.

Confidential: For Review Only

Table 6 Multiple regression analysis of significant factors of total cost and hospitalization cost

Variables	Hospitalization cost		Total Cost[a]	
	Coefficient (95% CI)[b]	P-value[b]	Coefficient (95% CI)[b]	P-value[b]
**Region (Shanghai[c])**				
Zhengzhou	0. 86（0. 7，1. 02）	<0. 01	0. 80（0. 67，0. 94）	<0. 01
Kunming	0. 45（0. 27，0. 63）	<0. 01	0. 63（0. 48，0. 78）	<0. 01
**Insurance status**	-0. 25（-0. 35，-0. 14）	<0. 01	-0. 18（-0. 27，-0. 09）	<0. 01
**Employment Status**				
**(Enterprise/self-employed[c])**				
Civil servants/ Public Institution	-0. 19（-0. 33，-0. 05）	0. 01	-0. 14（-0. 26，-0. 03）	0. 02
Full-time housewife	0. 03（-0. 14，0. 19）	0. 76	-0. 05（-0. 19，0. 09）	0. 46
Retirement	-0. 11（-0. 39，0. 17）	0. 44	-0. 18（-0. 41，0. 06）	0. 14
peasants	-0. 26（-0. 51，-0. 01）	0. 04	-0. 15（-0. 35，0. 06）	0. 17
Temporary workers	-0. 43（-0. 66，-0. 19）	<0. 01	-0. 26（-0. 45，-0. 06）	0. 01
Unemployment	-0. 02（-0. 29，0. 25）	0. 89	-0. 11（-0. 34，0. 11）	0. 32
**Hospitalization days**	0. 09（0. 08，0. 10）	<0. 01	0. 08（0. 07，0. 09）	<0. 01

Abbreviations: CI, confidence interval.

[a]Total cost includes hospitalization cost, traffic cost and indirect cost.

[b]Calculated using multiple linear regression.

[c]Reference category.

Confidential: For Review Only

References

1. Lu Y-Y, Luo R, Fu Z. Pathogen distribution and bacterial resistance in children with severe community-acquired pneumonia. Zhongguo dang dai er ke za zhi = Chinese journal of contemporary pediatrics. 2017;19(9):983-8.

2. McAllister DA, Liu L, Shi T, Chu Y, Reed C, Burrows J, et al. Global, regional, and national estimates of pneumonia morbidity and mortality in children younger than 5 years between 2000 and 2015: a systematic analysis. Lancet Glob Health. 2019;7(1):e47-e57.

3. Feng J, Yuan X, Zhu J, Li X, Miao L, He C, et al. Under-5-mortality rate and causes of death in China, 2000 to 2010. Chinese Journal of Epidemiology. 2012;33(6):558-61.

4. Song P, Theodoratou E, Li X, Liu L, Chu Y, Black RE, et al. Causes of death in children younger than five years in China in 2015: an updated analysis. Journal of Global Health. 2016;6(2).

5. Johnson J, Wilson KM, Zhou C, Johnson DP, Kenyon CC, Tieder JS, et al. Home Smoke Exposure and Health-Related Quality of Life in Children with Acute Respiratory Illness. Journal of Hospital Medicine. 2019;14(4):212-7.

6. Ma Z. Application effects of clinical nursing pathway in children with severe pneumonia. Medical Journal of Chinese People's Health. 2020;32(24):150-1+4.

7. Niu D, Chen L, Li J. Application of safety warning nursing mode in the whole course of nursing for children with severe pneumonia. Journal of Clinical Psychosomatic Diseases. 2020;26(01):186-9.

8. Hadning I, Andayani TM, Endarti D, Triasih R. Health-Related Quality of Life Among Children With Pneumonia in Indonesia Using the EuroQoL Descriptive System Value Set for

Confidential: For Review Only

Indonesia. Value Health Reg Issues. 2021;24:12-6.

9. Kulpeng W, Sornsrivichai V, Chongsuvivatwong V, Rattanavipapong W, Leelahavarong P, Cairns J, et al. Variation of health-related quality of life assessed by caregivers and patients affected by severe childhood infections. BMC Pediatr. 2013;13:122.

10. Wang S, Zhang T, Zhang X, Li H, Shi Y, Wang Y. Study on the prevalence characteristics and economic burden of acute respiratory diseases in children under 5 years old in Gansu. Maternal and Child Health Care of China. 2020;35(14):2548-52.

11. Wang J, Huang Z, Hu J, Jiang M, Lu J, Xu z, et al. Retrospective study on incidence and diagnosis of pneumonia in children under 5 years old in a community of Shanghai. Shanghai Journal of Preventive Medicine. 2018;30(7):552-6.

12. Zhou W, Shen A, Yang Z, Wang P, Wu B, Herdman M, et al. Patient-caregiver agreement and test-retest reliability of the EQ-5D-Y-3L and EQ-5D-Y-5L in paediatric patients with haematological malignancies. European Journal of Health Economics. 2021;22(7):1103-13.

13. Pan CW, Zhang RY, Luo N, He JY, Liu RJ, Ying XH, et al. How the EQ-5D utilities are derived matters in Chinese diabetes patients: a comparison based on different EQ-5D scoring functions for China. Qual Life Res. 2020;29(11):3087-94.

14. Zheng Y, Jit M, Wu JT, Yang J, Leung K, Liao Q, et al. Economic costs and health-related quality of life for hand, foot and mouth disease (HFMD) patients in China. Plos One. 2017;12(9).

15. Jiao F, Wong CKH, Gangwani R, Tan KCB, Tang SCW, Lam CLK. Health-related quality of life and health preference of Chinese patients with diabetes mellitus managed in primary

Confidential: For Review Only

care and secondary care setting: decrements associated with individual complication and number of complications. Health Qual Life Outcomes. 2017;15(1):125.

16. Liang W, Liu Y, Ren J. Observation on Efficacy of Cefixime Granules Combined with Ambroxol Oral Liquid in Treatment of Outpatient Pediatric Pneumonia. Evaluation and Analysis of Drug-Use in Hospitals of China. 2019;19(02):192-4.

17. Zhuge Y, Qian H, Zheng X, Huang C, Zhang Y, Zhang M, et al. Residential risk factors for childhood pneumonia: A cross-sectional study in eight cities of China. Environ Int. 2018;116:83-91.

18. Shoham Y, Dagan R, Givon-Lavi N, Liss Z, Shagan T, Zamir O, et al. Community-acquired pneumonia in children: quantifying the burden on patients and their families including decrease in quality of life. Pediatrics. 2005;115(5):1213-9.

19. Peng X. Hospitalization Cost Analysis of Common Infectious Diseases in Children from the Perspective of DRG [硕士]: China Medical University; 2022.

20. Wang T, Zheng Q, Wu Y, Yao Y, Kuang C, Li X. Investigation of economic burden caused by varicella in school aged children in Shenzhen. Disease Surveillance. 2021;36(10):1092-5.

21. Adamu AL, Karia B, Bello MM, Jahun MG, Gambo S, Ojal J, et al. The cost of illness for childhood clinical pneumonia and invasive pneumococcal disease in Nigeria. Bmj Global Health. 2022;7(1).

22. Hu J, Xu B, Shen K. Hospitalization Cost and Influencing Factors of Pneumonia with Children in a Certain Hospital in Beijing. Chinese Journal of Health Informatics and Management. 2018;15(2):211-6.

Confidential: For Review Only

Confidential: For Review Only

BMJ Paediatrics Open

Health-related quality of life and economic burden of childhood pneumonia in China: a multi-region study

Journal:	*BMJ Paediatrics Open*
Manuscript ID	bmjpo-2023-002031.R1
Article Type:	Original research
Date Submitted by the Author:	12-Jul-2023
Complete List of Authors:	Gao, Junyang; Fudan University<br>Fan, Jingzhi; Fudan University<br>Zhou, Huijun; University of Shanghai for Science and Technology<br>Jit, Mark; London School of Hygiene & Tropical Medicine<br>Wang, Pei; Fudan University
Keywords:	Health services research, Adolescent Health

SCHOLARONE™
Manuscripts

I, the Submitting Author has the right to grant and does grant on behalf of all authors of the Work (as defined in the below author licence), an exclusive licence and/or a non-exclusive licence for contributions from authors who are: i) UK Crown employees; ii) where BMJ has agreed a CC-BY licence shall apply, and/or iii) in accordance with the terms applicable for US Federal Government officers or employees acting as part of their official duties; on a worldwide, perpetual, irrevocable, royalty-free basis to BMJ Publishing Group Ltd ("BMJ") its licensees and where the relevant Journal is co-owned by BMJ to the co-owners of the Journal, to publish the Work in this journal and any other BMJ products and to exploit all rights, as set out in our licence.

The Submitting Author accepts and understands that any supply made under these terms is made by BMJ to the Submitting Author unless you are acting as an employee on behalf of your employer or a postgraduate student of an affiliated institution which is paying any applicable article publishing charge ("APC") for Open Access articles. Where the Submitting Author wishes to make the Work available on an Open Access basis (and intends to pay the relevant APC), the terms of reuse of such Open Access shall be governed by a Creative Commons licence – details of these licences and which Creative Commons licence will apply to this Work are set out in our licence referred to above.

Other than as permitted in any relevant BMJ Author's Self Archiving Policies, I confirm this Work has not been accepted for publication elsewhere, is not being considered for publication elsewhere and does not duplicate material already published. I confirm all authors consent to publication of this Work and authorise the granting of this licence.

Confidential: For Review Only

Health-related quality of life and economic burden of childhood pneumonia in China: a multi-region study

Junyang Gao [1,2*], BSc

Jingzhi Fan [1,2*], BSc

Huijun Zhou[3], PhD

Mark Jit[4], PhD

Pei Wang[1,2], PhD

[1] School of Public Health, Fudan University, Shanghai 200032, China

[2] Key Lab of Health Technology Assessment, National Health Commission of the People's Republic of China (Fudan University), Shanghai, China

[3] Business School, University of Shanghai for Science and Technology, Shanghai, China

[4] Department of Infectious Disease Epidemiology, Faculty of Epidemiology and Population Health, London School of Hygiene & Tropical Medicine, London, United Kingdom.

[*] The authors contribute equally

Corresponding to:

Pei Wang,

School of Public Health,

Fudan University

130 Dong An Road, Shanghai 200032, China.

E-mail: wang_p@fudan.edu.cn.

Text word count: 3555 words

Keywords: pneumonia, child, health-related quality of life, economic burden

Confidential: For Review Only

Abstract

Objective: To systematically investigate the health-related quality of life (HRQOL) and economic burden of children with pneumonia in different regions of China.

Study design: The study recruited a series of children under 5 years hospitalized for pneumonia in Shanghai, Zhengzhou, and Kunming from January to October 2019.

Health utility was assessed using the proxy version of EQ-5D-Y by interviewing patients' guardians face-to-face. The assessment was administered twice at patients' admission and discharge. Cost incurred for receiving the hospitalization were collected. Multiple linear regression and quantile regression were used to explore factors of EQ-5D-Y health utility score (HUS) and costs, respectively.

Results: A total of 501 pediatric patients with a median age (interquartile range, IQR) of 1.5 (0.83-2.71) years were included in the analysis. The mean HUS (standard deviations, SD) of the patients was 0.78 (0.18) at admission, and increased to 0.96 (0.10) at discharge. Some patients (14.2%) still felt worried, sad or unhappy after hospitalization. The mean hospitalization cost and total cost were RMB 5,859 (773 Euros) and RMB 6,439, respectively. The HUS was lower and the economic burden was heavier for the children in Zhengzhou. Apart from region, type of work, insurance status and hospital days were also related to the baseline HUS or HUS increment after treatment; insurance status, VAS score at discharge, guardians' employment and hospitalization days were associated with the costs.

Conclusion: The children with pneumonia have poor baseline HRQOL, and many of them still have mental health problems after treatment. The economic burden varied significantly across regions and is heavy for the patients' families in less developed areas (i.e., Zhengzhou and Kunming).

Keywords: pneumonia, child, health-related quality of life, economic burden

Confidential: For Review Only

Key message

Many studies have found that children with pneumonia had poorer health-related quality of life (HRQOL), especially in social and psychological functioning dimensions; pneumonia also placed a heavy economic burden on the children's family.

Previous studies did not provide health utility score (HUS) for Chinese children with pneumonia and were based on a single place. This study addressed the two issues by providing the HUS data, and comparing HRQOL and economic burden among different regions in China.

We found that many pediatric patients still had mental health problems after treatment, which deserve more attention in the post-discharge period. We also identified significant regional differences in HRQOL and economic burden, as well as other influencing factors.

Confidential: For Review Only

Introduction

Pneumonia is a significant type of lower respiratory tract infection affecting children worldwide(1), with serious impact on both physical and psychological aspects of health, such as coughing, breathlessness and irritability(2). It is the major single cause of death in children, causing approximately 1 million deaths annually, or 15% of an estimated 6.3 million deaths in children aged under 5 years(3). Studies have also shown that pneumonia is one of the top 5 causes of death in children under 5 years in China, accounting for 12.4% of deaths(4, 5). Pneumonia causes not only physical impairment, but also psychological, debilitating and social adjustment problems to the affected child. Hence, health-related quality of life (HRQOL), a comprehensive health outcome measure is required to holistically reflect the disease influence. Moreover, the HRQOL information could also be translated into health utility score (HUS) reflecting the value of HRQOL for use in economic evaluation if it is measured by utility instruments such as EQ-5D.

International and domestic studies have assessed HRQOL of pediatric patients with pneumonia using HRQOL instruments such as the Children's Quality of Life Scale (PQL 4.0)(6, 7) and the Generic Quality of Life Inventory-74 (GQOLI-74)(8). They found that the pediatric patients had poorer HRQOL, especially in social and psychological functioning dimensions. Studies in Indonesia and Thailand have also reported HUS of the patients using the proxy version of EQ-5D(9, 10). However, no studies have yet provided available data on HUS for the Chinese pediatric patients.

In China, some kinds of the costs incurred from pneumonia treatment, such as tests, anti-infective drugs, and bed charges, are not fully reimbursed according to Chinese healthcare system. Patients thus have to pay for the excess costs themselves. As a result, pneumonia also places a heavy financial burden on the children's family. Several studies have assessed its economic burden in childhood respiratory infections and pneumonia in China(11, 12). Wang et al. investigated the average length of stay (LOS) in hospital and hospitalization cost of 8,334 children with acute respiratory

Confidential: For Review Only

disease under 5 years in Gansu province from 2015 to 2018. They found that the LOS was 6.6 days and the expenditure was RMB 5,613 exceeding 30% of the per capita disposable income of the province in 2018. Similarly, Wang et al. conducted a retrospective study of 86 children with pneumonia under 5 years in a community in Shanghai during 2012. The study indicated the average total cost including outpatient cost, hospitalization cost, out-of-pocket drug cost, traffic cost, lost work cost was RMB 4,017, which accounted for 10% of the per capita disposable income of Shanghai in 2012. However, those studies were based on the patients in a single place, which may not be representative of the broader Chinese population and thus lead to limited extrapolation.

Hence, this study aimed to systematically investigate the HRQOL and economic burden of children with pneumonia from three different regions in China with divergent economic development levels.

Methods

Study design and patients

This study measured HRQOL of hospitalized pneumonia children at admission and discharge. Various costs associated with hospitalization were collected and aggregated. The study was conducted from January to October 2019 in three provincial capitals of sampled provinces representing different socio-economics statuses. The cities were Shanghai (eastern region), Zhengzhou (central region), and Kunming (western region), representing high, medium, and low status, respectively. In each city, a general or special (e.g., children's hospital) tertiary hospital was selected as the sampling hospital.

The inclusion criteria for the study were 1) younger than five years old, 2) clinical diagnosis of pneumonia, and 3) no concomitant diseases. All the pediatric patients hospitalized in the studying sites at the time of study were assessed for the eligibility by trained interviewers. Once a patient was eligible, his/her parents/guardians were invited to participate in the study.

Confidential: For Review Only

The consenting parents/guardians were interviewed face-to-face twice. The first interview was conducted within the first two days of patients' admission. A questionnaire was used to assess the child's HRQOL and to collect information on the child's (i.e., gender, age and medical insurance) and his/her guardian's sociodemographic characteristics (i.e., gender, age, education level, marriage status, employment, and monthly income level, race, domicile, religion, relationship with the child). The second interview was performed when the child was discharged from the hospital, and assessed the child's HRQOL again, as well as the total hospitalization costs including any household expenses, insurance payments (if any), lost work time, and the length of stay (LOS).

The study was supported by grants from the National Institute for Health Research (NIHR) (grant no. 16/137/109) using UK aid from the UK Government to support global health research. The views expressed in this publication are those of the author(s) and not necessarily those of the NIHR or the UK Department of Health and Social Care.

Health-related quality-of-life Measurement

The EQ-5D-Y is used as the HRQOL measure in the study. It is the youth version of the widely used EQ-5D that is more aligned with children's perceptions and understanding. It has five dimensions each with five response options: *walking about*, *looking after myself*, *doing usual activity*, *having pain or discomfort*, *feeling worried, sad or unhappy*.

The five response options of each dimension of Y-5L are *no problems* (level 1), *a little bit of problems* (level 2), *some problems* (level 3), *a lot of problems* (level 4), *extreme problems* (level 5)(13). As a result, it defines a total of 3,125 (5^5) health states by combing the responses of each dimension. A HUS can be assigned to each health state using a utility value set. Since the utility value set for the Y-5L is not available currently, the study used the Chinese value set of the EQ-5D-5L to calculate the HUS for the Y-5L health states(14). In addition, Y-5L uses a visual analog scale

https://mc.manuscriptcentral.com/bmjpo

Confidential: For Review Only

(VAS) with a score of 100 at the top for "the best imaginable health" and 0 at the bottom for "the worst imaginable health".

Given that the children aged 0-5 years were cared by their parents, it was assumed that the dimension “looking after myself” is irrelevant. For the children under 18 months who were unable to stand, the dimension “walking about” is assumed irrelevant(15, 16).

Economic Burden of Pneumonia

The economic burden of pneumonia was estimated by calculating direct medial cost (i.e., hospitalization cost including out-of-pocket cost, insurance payments if covered by insurance), non-medical cost (i.e., traffic cost), as well as indirect cost. Indirect cost was derived by estimating the loss of productivity of guardian using human capital approach. That is, the loss of work hour multiplies hourly wages based on annual average income in China in 2021 (8 hour per work day, and 250 work days per year). All cost data was adjusted to 2021 Chinese CNY using the latest published CPI index in China.

Statistical analysis

Descriptive statistical analyses were adopted to depict sociodemographic characteristics of the patients and their guardians, distributions of Y-5L dimension responses, Y-5L HUS and VAS score, as well as economic burden data. Categorical variables were presented using number and percentages; continuous variables were described using mean, standard deviations (SD) and range; and age is described using median and interquartile range (IQR). Chi-square test, median test or ANOVA test were used to compare the characteristics of the patients/guardians, HRQOL and economic burden of the patient among the three cities, and HRQOL between admission and discharge whenever appropriate. Comparison of HUS, VAS score and cost using the Tukey post hoc test(17) between samples were further conducted to determine which pairwise groups were different.

https://mc.manuscriptcentral.com/bmjpo

Multiple linear regression was used to analyze the factors influencing the HUS of children with pneumonia. HUS at baseline and its difference between baseline and discharge were used as the two dependent variables. According to previous studies(18, 19), hospitalization days and demographic characteristics including region, gender, age, insurance status of the children; and education level, employment of the guardians may have a significant effect on HUS. These factors were adopted as the independent variables in the two models (hospital days was adopted only in the model for HUS difference).

For the economic burden, quantile regression was used to analyze the impact factors. Referring to the previous studies (20, 21), three quantile points of 10%, 50% and 90% of hospitalization cost and total cost (hospitalization cost, transportation cost, and cost for productivity loss) were selected separately for the quantile regression analysis. The two costs were log-transformed to approximate normal distributions as the two dependent variables. Based on the evidence of prior studies(11, 12, 22, 23), gender, age, hospital days, insurance status, and disease prognosis may have a significant impact on costs and adopted as independent variables in the two models. Also, VAS score reflecting the disease prognosis, region and employment of guardians were also adopted in the models.

SPSS23.0 and SPSS 26.0 was used for all the analysis, and the threshold for significant differences was a P-value less than 0.05.

Results

Characteristics of study subjects

Table 1 shows the sociodemographic characteristics of children with pneumonia and their guardians. A total of 501 patients were included in analysis: 173, 120 and 208 in Shanghai, Zhengzhou and Kunming, respectively. The median age (IQR) of the patients was 1.50 (0.83-2.71) years, and the proportions of boys and girls were close (51.5% vs. 48.5%). A relatively high percentage of the children were without

Confidential: For Review Only

medical insurance (48.5%). More than half of the guardians were female (53.9%) with a median age (IQR) of 31.00 (28.00-35.00) years, and the majority of them were parents of the children (91.6%). They were mostly Han Chinese (90.6%), non-religious (96.8%), married (98.0%), from urban area (67.5%), university-educated or above (42.5%), enterprise/self-employed (41.9%). Most of them (57.8%) had family monthly income over 10,000 CNY (1319 Euros). The characteristics of the patients and their guardians differed among the three regions except for marital status (P=0.25).

Health-related quality-of-life of the children

Among the 501 patients, 394 patients responded to the EQ-5D-Y scale at admission, and 372 patients responded again at discharge. The percentage of reporting problems in the Y-5L dimensions at admission and discharge is shown in Table 2. At admission, the children had more problems in *having pain or discomfort* (85.5%) and *feeling sad, worried, or unhappy* (83.0%) dimensions; and the children in Zhengzhou had the highest prevalence of problems in each dimension. At discharge, the children's health status was significantly improved and no cases had very severe problems (level 5) in all the dimensions, while a relatively high proportion of problems was still observed in *feeling worried, sad or unhappy* dimension (19.0%). Similarly, the children in Zhengzhou tended to have more problems.

Table 3 reports the Y-5L HUS and VAS score of children with pneumonia in the three locations. The mean HUS of the patients at admission was 0.78, and the patients in Shanghai and Zhengzhou reported the highest (0.84) and lowest HUS (0.66), respectively. The HUS at discharge was significantly increased with the mean value being 0.96, and the children from Kunming and Zhengzhou had higher (0.99) or lower (0.90) HUS. The results of multiple comparisons showed significant difference in HUS between Shanghai and Zhengzhou, Kunming and Zhengzhou at both admission and discharge, while the difference between Shanghai and Kunming was insignificant. The difference in HUS at admission and discharge for children in

https://mc.manuscriptcentral.com/bmjpo

Zhengzhou was significantly greater than the difference in the other two regions. The mean VAS score of children in the three sites at both admission and discharge was also significantly different (62.9 vs. 89.2, P<0.05), with the children in Kunming having the highest score at both admission (65.4) and discharge (91.9), those in Zhengzhou and Shanghai having the lowest score at admission (58.3) and at discharge (77.2), respectively

Economic Burden of Pneumonia

The economic burden manifested by hospitalization cost and total cost summing of hospitalization cost, transportation cost, and indirect cost are shown in Table 4. The mean hospitalization cost of the three regions was RMB 5,859 and the children in Zhengzhou (RMB 8,667) had much higher cost than that in Shanghai (RMB 3,100) and Kunming (RMB 6,522). The mean transportation cost was RMB 110, and the children in Kunming (RMB 220) had the highest cost followed by the children in Shanghai (RMB 4) and Zhengzhou (RMB 73). The average hospitalization days was 8.0 days: the days were 10.6 days, 8.2 days, and 4.8 days for the children in Kunming, Shanghai, and Zhengzhou, respectively. Correspondingly, the average time of loss work of guardians in Kunming (65.9 hours) was substantially higher than that in Shanghai (6.5 hours) and Zhengzhou (33.1 hours). This also resulted in the highest indirect cost in Kunming (RMB 710), followed by Zhengzhou (RMB 444), and Shanghai (RMB 187). For the total cost, the average amount was RMB 6,439, and the patients in Zhengzhou had the highest amount (RMB 9,183), followed by Kunming (RMB 7,464), and Shanghai (RMB 3,293).

Regression analyses

Table 5 present the significant coefficients generated from the multiple linear regressions. Region, insurance status and type of work were significantly associated with baseline HUS (Table 5). That is, the children in Zhengzhou, without insurance, and their guardians who were temporary workers had lower baseline HUS. Region and hospitalization days were significantly correlated with the difference in HUS: the

Confidential: For Review Only

children in Zhengzhou and with longer hospital days were more likely to have higher difference in HUS (Table 5).

The results of quantile regression analysis showed that region, insurance status, hospital days, VAS score at discharge and employment of guardians were significantly associated with both hospitalization and total costs (Table 6). Specifically, region had a significant effect at almost all quartiles, and the children in Zhengzhou and Kunming had higher costs. In addition, the degree of the effect was higher at the lower quartile than at the higher quartile for children in Zhengzhou. The two costs were significantly lower for the children without insurance and with shorter hospitalization days at the middle and high quartiles, and the effects of hospitalization days were stronger in the higher quartile. For children with higher VAS score at discharge, the two costs were significantly higher at the low and middle quantiles. At the low quantile, the two costs were significantly lower for children whose guardians were civil servant or public institution worker, and total cost was also lower for the children with retired working guardians. For children with guardians who were temporary workers, the two costs were significantly lower at almost all quartiles.

Discussion

We found that the children with pneumonia had inferior health status both physically and psychologically; and the disease economic burden varied greatly among regions. Moreover, we also identified several important factors influencing the HUS and economic burden. Therefore, this study deepens the understanding of HRQOL and economic burden of the children in China.

At admission, the majority of children had health problems especially in *having pain or discomfort*, *feeling worried*, *sad or unhappy* dimensions as well as low HUS and VAS score. Previous studies have also reported similar findings that the pediatric patients with pneumonia had poor HRQOL at admission, especially in social and psychological functioning dimensions(6, 7). When pneumonia occurred, the children

Confidential: For Review Only

had symptoms such as fever, cough, sore throat and breathlessness(24), which may bring physical discomfort to the children, affect their emotions and lead to psychological problems. We further found that although their health improved a lot at discharge, many of them were still feeling worried, sad, or unhappy at discharge. Thus, their psychological condition should deserve more attention in the post-discharge period.

Among the three regions, the children in Zhengzhou had worse baseline HUS, which was probably due to that they were mainly from urban area containing more risk factors that may contribute to the development of pneumonia in childhood(25). The children without insurance also reported poorer baseline HUS. Some studies revealed that individuals who have health insurance reported a higher quality of life than those without health insurance(26, 27). In addition, the children also had poorer baseline HUS when their guardians were temporary workers. An Israeli study showed that both parental unemployment and workday loss affected the HRQOL of children with pneumonia as well(28). Temporary work means unstable income, making it difficult to provide good care to the children. The children in Zhengzhou had larger HUS difference. Since the patients were discharged from hospitals because their health was recovered and their HUS at discharge was close to full health, the identified association could be attributed to the HUS at baseline. That is, the children in Zhengzhou had worse baseline HUS. In addition, children with longer hospital days had higher HUS difference, probably because longer treatment time corresponds to better treatment effect.

We found that the average hospitalization and total costs were comparable to the previous results for the Chinese children under 5 years with pneumonia(11, 12): the average hospitalization cost was RMB 5,771 in Gansu and the average total cost was RMB 4,642 in Shanghai after being adjusted for 2021 CNY. The costs were also similar to the costs of other common childhood infectious diseases in China. According to 2021 CNY, the average hospitalization costs for children in Shenyang with scarlet fever and hand, foot and mouth disease were RMB 4,979 and RMB 5,050,

Confidential: For Review Only

respectively(29); the average total cost for inpatient cases with chickenpox in school-age children was RMB 3,312 in Shenzhen(30). On the other hand, we identified the disease economic burden varied greatly among the regions: the total cost was calculated to be 1.9% of the local per capita GDP in Shanghai, 9.6% in Zhengzhou, and 8.7% in Kunming, respectively. The finding indicated that the disease places severer economic burden on the children's families in less economically developed areas. The government thus could take necessary activities (e.g., increasing the reimbursement rate of medical insurance) to alleviate the burden of the families.

The children in Zhengzhou had higher hospitalization cost. This may be due to their worse baseline health status, requiring more medical resources and thus higher costs. For the transportation and indirect costs, the patients in Kunming had higher amount. Kunming is the capital city of Yunnan province, which had a relatively low level of socio-economic development and a lack of medical resources compared to other regions of China. Hence, the patients in other cities of the province have to concentrate in Kunming, leading to the higher costs.

The children without medical insurance had significantly lower hospitalization and total costs. A previous study had indicated that relying solely on out-of-pocket payment provided obstacles for treatment access, skewing treatment seeking towards those affordable(23), leading to lower costs. The two costs were also significantly associated with hospitalization days, and similar findings had been reported in a previous study that shortening the number of ineffective hospitalization days helped to reduce the financial burden(22). In the study, we found that children with higher VAS score at discharge had higher hospitalization and total costs. This may be because achieving better health required more consumption of medical services. We also found that the two costs were significantly higher for the children whose guardians were enterprise or self-employed workers. This was probably because they may have better economic conditions and were willing to spend more for their children to get better treatment.

https://mc.manuscriptcentral.com/bmjpo

Confidential: For Review Only

This study has several limitations which should be noted. First, the study was conducted in three areas in China, which may not be representative of the entire country or other regions with different socioeconomic statuses. Also, only pediatric patients who were hospitalized at the time of the study were included; while those who were managed on an outpatient basis or treated at primary care clinics were not included. In addition, there were missing values in the responses to the EQ-5D-Y dimensions for the patients in Shanghai. These may have limited the representativeness of the study population and affected the generalizability of our findings. Second, due to the study design and resources, we did not enroll a control group of children without pneumonia for comparison. Thus, it may be difficult to attribute the observed results exclusively to pneumonia. Third, the Y-5L was designed mainly for children over 6 years of age, and the reliability and validity of its proxy version need to be further tested when it is applied to the guardians of children under 5 years. Also, the Chinese Y-5L value set has not been available, so the Chinese EQ-5D-5L value set was used to calculate the HUS instead. This introduces some uncertainty in the accuracy of the HRQOL measurements. Fourth, we did not collect certain clinical characteristics including disease severity and treatment modality, thus their influence on HRQOL and economic burden cannot be assessed. Fifth, although we excluded the pneumonia patients with other acute diseases, there may be undiagnosed comorbidities during data collection, which may affect HRQOL scores. Last but not least, the study lacked long-term follow-up, thus it was not possible to know the trajectory of HRQOL and economic burden over time.

Conclusion

The pediatric patients with pneumonia mainly suffered from pain, discomfort and felt sad, worried, or unhappy at admission, and many of them still had mental health problems after treatment. Region, insurance status, hospitalization days and employment of guardians were the four factors influencing their HRQOL. The economic burden of the disease varied significantly across regions and was heavy for

Confidential: For Review Only

the patients' families in less developed areas. Insurance status of the patients, employment status of their guardians, VAS score at discharge and hospitalization days were also associated with the burden.

Acknowledgements: We gratefully acknowledge the study participants and their families. We thank the hospitals for their support and cooperation with our data collection.

Contributors: JYG: Methodology, data analysis, manuscript writing (original draft). JZF: Data analysis, manuscript writing (original draft and revision). PW: Conceptualization, methodology, critical manuscript revision, supervision and project administration. All authors critically revised the manuscript and approved the final version of manuscript.

Funding: This research was funded by the National Institute for Health Research (NIHR) (GHR 16/137/09) using UK aid from the UK Government to support global health research. The views expressed in this publication are those of the author(s) and not necessarily those of the NIHR or the UK government.

Ethic: This study was conducted according to the guidelines of the Declaration of Helsinki and approved by the Institutional Review Board of School of Public Health, Fudan University (IRB00002408).

Competing interests: The authors declare no competing interests

Patient and public involvement: Patients and/or the public were not involved in the design, conduct, reporting or dissemination plans of this research

Provenance and peer review: Not commissioned; externally peer reviewed.

Data availability: The datasets analyzed during the current study are available from the corresponding author on reasonable request

Confidential: For Review Only

Confidential: For Review Only

Table 1 Sociodemographic characteristics of children with pneumonia and their guardians among the three regions

Variables	Overall N=501	Shanghai N=173	Zhengzhou N=120	Kunming N=208	P-value[a]
**Characteristics of the child**					
**Gender**					< 0.01
Boys	258 (51.5)	72 (41.6)	63 (52.5)	123 (59.1)	
Girls	243 (48.5)	101 (58.4)	57 (47.5)	85 (40.9)	
**Age(year)**					< 0.01
Median	1.50	1.00	2.50	1.17	
IQR	0.83-2.71	0.92-2.00	2.08-3.50	0.40-2.33	
**Medical Insurance**					< 0.01
Yes	185 (36.9)	91 (52.6)	41 (34.2)	53 (25.5)	
None	243 (48.5)	80 (46.2)	79 (65.8)	84 (40.4)	
Unknown	71 (14.2)	-	-	71 (34.1)	
**Guardianship characteristics**					
**Age(year)**					0.04
Mean (SD)	31.00	31.00	32.50	31.00	
Range	28.00-35.00	28.00-37.00	30.00-35.00	27.00-34.00	
**Gender**					< 0.01
Male	209 (41.7)	57 (32.9)	42 (35.0)	110 (52.9)	
Female	292 (58.3)	116 (67.1)	78 (65.0)	98 (47.1)	
**Relationship**					< 0.01
Father	189 (37.7)	35 (20.2)	42 (35.0)	112 (53.8)	
Mother	270 (53.9)	105 (60.7)	78 (65.0)	87 (41.8)	
Other[b]	42 (8.4)	33 (19.1)	-	9 (4.3)	
**Ethnic**					< 0.01
Han	454 (90.6)	171 (98.8)	118 (98.3)	165 (79.3)	
Others	47 (9.4)	2 (1.2)	2 (1.7)	43 (20.7)	
**Religion**					0.01
None	485 (96.8)	170 (98.3)	118 (98.3)	197 (94.7)	
Yes or refused to answer	13 (2.6)	3 (1.7)	2 (1.6)	11 (5.3)	
**Marriage Status**					0.25
Married	491 (98.0)	170 (98.3)	120 (100.0)	201 (96.6)	
Singles and others	10 (2.0)	3 (1.8)	-	7 (3.4)	
**Domicile**					< 0.01
City	241 (48.1)	102 (59.0)	95 (79.2)	44 (21.2)	
Town	97 (19.4)	43 (24.9)	20 (16.7)	34 (16.3)	
Rural	161 (32.1)	27 (15.6)	5 (4.2)	129 (62.0)	
**Education**					< 0.01
Elementary school and below	28 (5.6)	11 (6.3)	-	17 (8.2)	
Junior High School	104 (20.8)	14 (8.1)	2 (1.7)	88 (42.3)	
High School	88 (17.6)	30 (17.3)	15 (12.5)	43 (20.7)	

Confidential: For Review Only

College	68 (13.6)	23 (13.3)	25 (20.8)	20 (9.6)	
University and above	213 (42.5)	95 (54.9)	78 (75.0)	40 (19.2)	
**Employment**					< 0.01
Enterprise/self-employed	210 (41.9)	77 (44.5)	83 (69.2)	50 (24.0)	
Civil servants / Public Institution	83 (16.6)	20 (11.6)	24 (20.0)	39 (18.8)	
Full-time home/housewife	73 (14.6)	37 (21.4)	5 (4.2)	31 (14.9)	
Retirement	21 (4.2)	18 (10.4)	-	3 (1.4)	
Farming	50 (10.0)	11 (6.4)	3 (2.5)	36 (17.3)	
Temporary workers	39 (7.8)	1 (0.6)	4 (3.3)	34 (16.3)	
Unemployment	21 (4.2)	7 (4.1)	1 (0.8)	13 (6.2)	
Other or refused to answer	3 (0.6)	1 (0.6)	-	2 (1.0)	
**Family monthly income (CNY)**					< 0.01
<5000 (660Euros)	83 (16.6)	-	3 (2.5)	80 (38.5)	
5000-10000	97 (19.4)	-	20 (16.7)	77 (37.0)	
10000-20000	76 (15.2)	9 (5.2)	47 (39.2)	20 (9.6)	
20,000-30,000	75 (15.0)	37 (21.4)	34 (28.3)	4 (1.9)	
> 30,000	117 (23.4)	94 (54.3)	16 (13.3)	7 (3.3)	
Unknown or refused to answer	52 (10.4)	32 (18.5)	-	20 (9.6)	

IQR: interquartile range; SD: standard deviations; CNY: Chinese Yuan.

[a]Comparison of population characteristics among the three regions. Categorical variables and continuous variables were analyzed using chi-square test and ANOVA test, respectively.

[b]This refers to a guardian such as a non-parental relative of the affected child.

Confidential: For Review Only

Table 2 Guardians' responses to the EQ-5D-Y dimensions at admission and discharge

Dimension	Overall		Shanghai		Zhengzhou		Kunming		P-value[a]		
	Admission N=394	Discharge N=372	Admission N=66	Discharge N=44	Admission N=120	Discharge N=120	Admission N=208	Discharge N=208	Admission	Discharge	Comparison[b]
**Walking about**									<0.01	0.06	<0.01
No problems	152 (38.6%)	202 (54.3%)	59 (89.4%)	44 (100.0%)	27 (22.5%)	66 (55.0%)	66 (31.7%)	92 (44.2%)			
A little bit of problems	86 (21.8%)	32 (8.6%)	5 (7.6%)	0	56 (46.7%)	27 (22.5%)	25 (12.0%)	5 (2.4%)			
Some problems	16 (4.1%)	6 (1.6%)	0	0	10 (8.3%)	6 (5.0%)	6 (2.9%)	0			
A lot of problems	7 (1.8%)	2 (0.5%)	0	0	6 (5.0%)	1 (0.8%)	1 (0.5%)	1 (0.5%)			
Extreme problems	1 (0.3%)	0	0	0	1 (0.8%)	0	0	0			
＜18month[c]	132（33.5%）	130（34.9%）	2 (3.0%)	0	20 (16.7%)	20 (16.7%)	110 (52.9%)	110 (52.9%)			
**Doing usual activities**									<0.01	<0.01	<0.01
No problems	146 (37.1%)	319 (85.8%)	36 (54.5%)	43 (97.7%)	20 (16.7%)	77 (64.2)	90 (43.3%)	199 (95.7%)			
A little bit of problems	185 (47.0%)	45 (12.1%)	30 (45.5%)	1 (2.3%)	67 (55.8%)	35 (29.2)	88 (42.3%)	9 (4.3%)			
Some problems	46 (11.7%)	7 (1.9%)	0	0	23 (19.2%)	7 (5.8)	23 (11.1%)	0			
A lot of problems	11 (2.8%)	1 (0.3%)	0	0	7 (5.8%)	1 (0.8)	4 (1.9%)	0			
Extreme problems	6 (1.5%)	0	0	0	3 (2.5%)	0	3 (1.4%)	0			
**Having pain or discomfort**									<0.01	<0.01	<0.01
No problems	56 (14.2%)	318 (85.5%)	6 (9.1%)	40 (90.9%)	4 (3.3%)	79 (65.8%)	46 (22.1%)	199 (95.7%)			
A little bit of problems	223 (56.6%)	43 (11.6%)	54 (81.8%)	4 (9.1%)	60 (50.0%)	30 (25.0%)	109 (52.4%)	9 (4.3%)			
Some problems	93 (23.6%)	9 (2.4%)	6 (9.1%)	0	42 (35.0%)	9 (7.5%)	45 (21.6%)	0			
A lot of problems	20 (5.1%)	2 (0.5%)	0	0	12 (10.0%)	2 (1.7%)	8 (3.8%)	0			
Extreme problems	2 (0.5%)	0	0	0	2 (1.7%)	0	0	0			
**Feeling worried, sad or unhappy**									<0.01	<0.01	<0.01

Confidential: For Review Only

No problems	67 (17.0%)	301 (81.0%)	8 (12.1%)	22 (50.0%)	1 (0.8%)	78 (65.0%)	58 (27.9%)	201 (96.6%)
A little bit of problems	193 (49.0%)	63 (16.9%)	33 (50.0%)	22 (50.0%)	58 (48.3%)	34 (28.3%)	102 (49.0%)	7 (3.4%)
Some problems	101 (25.6%)	6 (1.6%)	25 (37.9%)	0	37 (30.8%)	6 (5.0%)	39 (18.8%)	0
A lot of problems	33 (8.4%)	2 (0.5%)	0	0	24 (20.0%)	2 (1.7%)	9 (4.3%)	0
Extreme problems	0	0	0	0	0	0	0	0

[a]Calculated using the chi-square test.

[b]The comparison of the responses of the guardians on EQ-5D-Y dimensions at admission and discharge.

[c]Children younger than 18 months were assumed to have no problem on walking about dimension.

Confidential: For Review Only

Table 3 EQ-5D-Y health utility and EQ-VAS scores of children with pneumonia in the three locations

	Total<br>Admission: N=394<br>Discharge: N=372	Shanghai (1)<br>Admission: N=66<br>Discharge: N=44	Zhengzhou (2)<br>Admission: N=120<br>Discharge: N=120	Kunming (3)<br>Admission: N=208<br>Discharge: N=208	Post hoc analysis[a]	P-value[b]
**Utility admission**						
Mean (SD)	0.78 (0.18)	0.84 (0.07)	0.66 (0.21)	0.82 (0.15)	(1) > (2); (3) > (2)	<0.001
Range	-0.04-1.00	0.63-1.00	-0.04-0.95	0.25-1.00		
**VAS admission**						
Mean (SD)	62. 9 (16. 6)	63. 5 (14. 2)	58. 3 (15. 5)	65. 4 (17. 4)	(3) > (2)	<0. 01
Range	15-100	40-95	15-84	20-100		
**Utility discharge**						
Mean (SD)	0. 96 (0. 10)	0. 97 (0. 04)	0. 90 (0. 15)	0. 99 (0. 04)	(1) > (2); (3) > (2)	<0.001
Range	0. 27-1. 00	0. 85-1. 00	0. 27-1. 00	0. 67-1. 00		
**VAS discharge**						
Mean (SD)	89. 2 (13. 0)	77. 2 (9. 5)	88. 9 (10. 7)	91. 9 (13. 5)	(2) > (1); (3) > (1)	<0. 01
Range	40-100	60-95	47-100	40-100		
**Utility difference**						
Mean (SD)	0. 19 (0. 16)	0. 15 (0. 06)	0. 24 (0. 21)	0. 17 (0. 14)	(1) < (2); (2) > (3)	<0.001
Range	-0. 05-1. 04	0. 04-0. 32	0-1. 04	-0. 05-0. 75		
**VAS difference**						
Mean (SD)	27. 1 (13. 5)	20. 1 (7. 7)	30. 6 (14. 3)	26. 5 (13. 4)	(2) > (1); (2) > (3)	<0. 01
Range	0-74	5-40	10-74	0-60		

SD, standard deviations.

[a]Comparison using the Tukey post hoc test.

[b]Calculated using ANOVA test.

Confidential: For Review Only

Table 4 Economic burden of disease in children with pneumonia

	Overall<br>N=501	Shanghai<br>(1)	Zhengzhou<br>(2)	Kunming<br>(3)	Post hoc comparison[a]	P value[b]
**Traffic cost**						<0.01
Mean (SD)	110.0（270.1）	4.3（9.8）	72.9（138.2）	219.9（378.6）	(1) < (3);	
Range	0-3514	0-60	0-780	0-3514	(2) < (3)	
**Out-of-pocket cost**						
Mean (SD)	3816.1(3981.9)	2036.6（1077.7）	3813.8(1990.3)	5229.1（5473.3）	(1) < (2) < (3)	<0.01
Range	471-60420	471-8236	1350-10416	504-60420		
**Hospitalization cost**					(1) < (2);	
Mean (SD)	5859.2（5486.5）	3099.7（1474.2）	8666.6(3965.2)	6521.5（7098.9）	(1) < (3);	<0.01
Range	302-80560	987-8261	3114-18998	302-80560	(2) > (3)	
**Loss of work time (hour)**						<0.01
Mean (SD)	37.9（81.1）	6.5（3.9）	33.1（15.8）	65.9（121.0）		
Range	0-1200	0-10	9-80	0-1200	–	
**Indirect cost[c]**						
Mean (SD)	465.79（967.89）	187.40（171.73）	443.50 (215.60)	710.20（1444.48）	(1) < (3);	<0.01
Range	0-15399.60	0-390.14	107.24-1072.44	0-15399.60	(2) < (3)	
**Hospitalization days**						<0.01
Mean (SD)	8.0（6.6）	4.8（1.9）	8.2（3.0）	10.6(9.1)	–	
Range	0-50	1-11	5-15	0-50		
**Total Cost[d]**						<0.01
Mean (SD)	6439.37（5772.99）	3292.52（1511.23）	9183.00（4085.72）	7463.64（7437.87）	(1) < (3) < (2)	
Range	918.09-80560.48	986.86-8701.53	3370.06-19346.78	918.09-80560.48		

SD, standard deviations.

[a]Comparison using the Tukey post hoc test.

[b]Calculated using ANOVA test.

[c]Indirect cost: loss of work hour multiples income per hour that converted from annual average income in China 2021 (8 hour per work day, and 250 work days per year).

[d]Total cost includes hospitalization cost, traffic cost and indirect cost.

Confidential: For Review Only

Table 5 Multiple regression analysis of baseline health utility score and health utility score difference between baseline and follow-up

Variables	Baseline HUS		HUS difference (discharge-admission)	
	Coefficient (95% CI)[a]	P-value[a]	Coefficient (95% CI)[a]	P-value[a]
**Region (Shanghai[b])**				
Zhengzhou	-0.17（-0.22, -0.12）	<0.01	0.06（0.01, 0.12）	0.02
Kunming	0.04（-0.01, 0.1）	0.12	-0.06（-0.12, 0）	0.06
**Gender**	-0.02（-0.05, 0.02）	0.32	0.01（-0.02, 0.04）	0.48
**Age**	0.002（-0.01, 0.02）	0.72	-0.003 (-0.02, 0.01)	0.63
**Insurance status**	-0.001（-0.001, 0）	0.01	0.001（0, 0.001）	0.06
**Education**				
**（Elementary school and below[b]）**				
Junior High School	-0.001（-0.09, 0.09）	0.98	0.00（-0.09, 0.09）	1.00
High School	-0.03（-0.09, 0.03）	0.29	0.02 (-0.05, 0.08）	0.64
College	-0.03（-0.09, 0.02）	0.20	0.04（-0.02, 0.09）	0.16
University and above	0.02（-0.03, 0.07）	0.43	-0.04（-0.09, 0.01）	0.11
**Employment Status**				
**(Enterprise/self-employed[b])**				
Civil servants / Public Institution	-0.02（-0.07, 0.02）	0.30	0.03（-0.02, 0.07）	0.21
Full-time home/housewife	0.03（-0.03, 0.09）	0.39	-0.03（-0.09, 0.03）	0.33
Retirement	0.06（-0.05, 0.16）	0.27	-0.03（-0.15, 0.09）	0.60
Farming	-0.03（-0.09, 0.03）	0.32	0.02（-0.04, 0.08）	0.50
Temporary workers	-0.08（-0.14, -0.02）	0.01	0.05（-0.01, 0.11）	0.08
Unemployment	-0.07（-0.15, 0.02）	0.13	0.04（-0.05, 0.13）	0.37
**Hospitalization days**	-	-	0.01（0.002, 0.01）	<0.01

Abbreviations: HUS, health utility score; CI, confidence interval.

[a]Calculated using multiple linear regression.

[b]Reference category.

Confidential: For Review Only

Table 6 Quantile regression analysis of total cost and hospitalization cost

Variables	Hospitalization cost			Total Cost[a]		
	q=0.1	q=0.5	q=0.9	q=0.1	q=0.5	q=0.9
**Region (Shanghai[b])**						
Zhengzhou	1.06**	0.91**	0.71**	0.96**	0.86**	0.74**
Kunming	0.35	0.56**	0.53**	0.62**	0.64**	0.66**
**Gender**	-0.03	-0.07	-0.01	-0.03	-0.07	-0.01
**Age**	-0.03	0.002	-0.01	0.002	0.01	-0.002
**Insurance status**	0.003	-0.003*	-0.004*	0.001	-0.004**	-0.004**
**VAS-discharge**	0.02**	0.01*	0.001	0.01**	0.01*	0.000
**Employment Status (Enterprise/self-employed[b])**						
Civil servants / Public Institution	-0.31*	-0.07	-0.04	-0.26**	-0.08	-0.04
Full-time home/housewife	-0.04	0.08	0.07	-0.12	0.03	0.04
Retirement	-0.40	-0.22	0.14	-0.56*	-0.36	0.04
Farming	-0.13	-0.05	-0.11	-0.03	-0.05	0.04
Temporary workers	-0.54*	-0.23*	-0.26*	-0.32*	-0.16	-0.27*
Unemployment	0.13	0.07	0.13	-0.08	0.05	0.03
**Hospitalization days**	0.01	0.02**	0.08**	0.01	0.02**	0.07**

[a]Total cost includes hospitalization cost, traffic cost and indirect cost.

[b]Reference category.

***:P<0.05, **:P<0.01**

Confidential: For Review Only

References

1. Lu Y-Y, Luo R, Fu Z. Pathogen distribution and bacterial resistance in children with severe community-acquired pneumonia. Zhongguo dang dai er ke za zhi = Chinese journal of contemporary pediatrics. 2017;19(9):983-8.

2. K W, P G, R P, A T, D M, A H. - Clinical symptoms and signs for the diagnosis of Mycoplasma pneumoniae in. D - 100909747. (- 1469-493X (Electronic)):- CD009175.

3. HJ Z, W B, L M, MP N. - Childhood pneumonia - the Drakenstein Child Health Study. D - 0404520. (- 0256-9574 (Print)):- 642-3.

4. Feng J, Yuan X, Zhu J, Li X, Miao L, He C, et al. Under-5-mortality rate and causes of death in China, 2000 to 2010. Chinese Journal of Epidemiology. 2012;33(6):558-61.

5. Song P, Theodoratou E, Li X, Liu L, Chu Y, Black RE, et al. Causes of death in children younger than five years in China in 2015: an updated analysis. Journal of Global Health. 2016;6(2).

6. Johnson J, Wilson KM, Zhou C, Johnson DP, Kenyon CC, Tieder JS, et al. Home Smoke Exposure and Health-Related Quality of Life in Children with Acute Respiratory Illness. Journal of Hospital Medicine. 2019;14(4):212-7.

7. Ma Z. Application effects of clinical nursing pathway in children with severe pneumonia. Medical Journal of Chinese People’s Health. 2020;32(24):150-1+4.

8. Niu D, Chen L, Li J. Application of safety warning nursing mode in the whole course of nursing for children with severe pneumonia. Journal of Clinical Psychosomatic Diseases. 2020;26(01):186-9.

9. Hadning I, Andayani TM, Endarti D, Triasih R. Health-Related Quality of Life Among Children With Pneumonia in Indonesia Using the EuroQoL Descriptive System Value Set for Indonesia. Value Health Reg Issues. 2021;24:12-6.

Confidential: For Review Only

10. Kulpeng W, Sornsrivichai V, Chongsuvivatwong V, Rattanavipapong W, Leelahavarong P, Cairns J, et al. Variation of health-related quality of life assessed by caregivers and patients affected by severe childhood infections. BMC Pediatr. 2013;13:122.

11. Wang S, Zhang T, Zhang X, Li H, Shi Y, Wang Y. Study on the prevalence characteristics and economic burden of acute respiratory diseases in children under 5 years old in Gansu. Maternal and Child Health Care of China. 2020;35(14):2548-52.

12. Wang J, Huang Z, Hu J, Jiang M, Lu J, Xu z, et al. Retrospective study on incidence and diagnosis of pneumonia in children under 5 years old in a community of Shanghai. Shanghai Journal of Preventive Medicine. 2018;30(7):552-6.

13. Zhou W, Shen A, Yang Z, Wang P, Wu B, Herdman M, et al. Patient-caregiver agreement and test-retest reliability of the EQ-5D-Y-3L and EQ-5D-Y-5L in paediatric patients with haematological malignancies. European Journal of Health Economics. 2021;22(7):1103-13.

14. Pan CW, Zhang RY, Luo N, He JY, Liu RJ, Ying XH, et al. How the EQ-5D utilities are derived matters in Chinese diabetes patients: a comparison based on different EQ-5D scoring functions for China. Qual Life Res. 2020;29(11):3087-94.

15. Zheng Y, Jit M, Wu JT, Yang J, Leung K, Liao Q, et al. Economic costs and health-related quality of life for hand, foot and mouth disease (HFMD) patients in China. Plos One. 2017;12(9).

16. JT W, M J, Y Z, K L, W X, J Y, et al. - Routine Pediatric Enterovirus 71 Vaccination in China: a Cost-Effectiveness. D - 101231360. (- 1549-1676 (Electronic)):- e1001975.

17. Jiao F, Wong CKH, Gangwani R, Tan KCB, Tang SCW, Lam CLK. Health-related quality of life and health preference of Chinese patients with diabetes mellitus managed in primary care and secondary care setting: decrements associated with individual complication and number of

Confidential: For Review Only

complications. Health Qual Life Outcomes. 2017;15(1):125.

18. MR T, R M-S, C Z, DR W, EE H, DP J, et al. - Obesity and Health-Related Quality of Life in Children Hospitalized for Acute. D - 101585349. (- 2154-1671 (Electronic)):- 841-8.

19. N M, GP P, SR H, T R, A U, MA P, et al. - Parents' Working Conditions in the Early COVID-19 Pandemic and Children's. D - 101304551. (- 1661-8564 (Electronic)):- 1605036.

20. MA O, F T, AE W, KB N, DK W, VJ F, et al. - Use of Quantile Regression to Determine the Impact on Total Health Care Costs of. D - 0372354. (- 1528-1140 (Electronic)):- 331-9.

21. Lei W, Yaya Y, Yini Z, Feng T, Xiaobin H. Analysis of influencing factors of hospitalizat ion expenses in elderly patients with injury based on quantile regress ion and random forest. Chinese Journal of Prevention and Control of Chronic Diseases. 2023;31(04):246-50.

22. Hu J, Xu B, Shen K. Hospitalization Cost and Influencing Factors of Pneumonia with Children in a Certain Hospital in Beijing. Chinese Journal of Health Informatics and Management. 2018;15(2):211-6.

23. Adamu AL, Karia B, Bello MM, Jahun MG, Gambo S, Ojal J, et al. The cost of illness for childhood clinical pneumonia and invasive pneumococcal disease in Nigeria. Bmj Global Health. 2022;7(1).

24. Liang W, Liu Y, Ren J. Observation on Efficacy of Cefixime Granules Combined with Ambroxol Oral Liquid in Treatment of Outpatient Pediatric Pneumonia. Evaluation and Analysis of Drug-Use in Hospitals of China. 2019;19(02):192-4.

25. Zhuge Y, Qian H, Zheng X, Huang C, Zhang Y, Zhang M, et al. Residential risk factors for childhood pneumonia: A cross-sectional study in eight cities of China. Environ Int. 2018;116:83-91.

26. M B, Thomas J r. - Health insurance coverage and health-related quality of life: analysis of 2000.

Confidential: For Review Only

D - 9103800. (- 1049-2089 (Print)):- 643-54.

27. RE G, L R, EG E, J D, L C. - Quality of life and associated characteristics in a large national sample of. D - 7805975. (- 0149-5992 (Print)):- 562-7.

28. Shoham Y, Dagan R, Givon-Lavi N, Liss Z, Shagan T, Zamir O, et al. Community-acquired pneumonia in children: quantifying the burden on patients and their families including decrease in quality of life. Pediatrics. 2005;115(5):1213-9.

29. Peng X. Hospitalization Cost Analysis of Common Infectious Diseases in Children from the Perspective of DRG [硕士]: China Medical University; 2022.

30. Wang T, Zheng Q, Wu Y, Yao Y, Kuang C, Li X. Investigation of economic burden caused by varicella in school aged children in Shenzhen. Disease Surveillance. 2021;36(10):1092-5.

Confidential: For Review Only

BMJ Paediatrics Open

Health-related quality of life and economic burden of childhood pneumonia in China: a multi-region study

Journal:	*BMJ Paediatrics Open*
Manuscript ID	bmjpo-2023-002031.R2
Article Type:	Original research
Date Submitted by the Author:	08-Aug-2023
Complete List of Authors:	Gao, Junyang; Fudan University<br>Fan, Jingzhi; Fudan University<br>Zhou, Huijun; University of Shanghai for Science and Technology<br>Jit, Mark; London School of Hygiene & Tropical Medicine<br>Wang, Pei; Fudan University
Keywords:	Health services research, Adolescent Health

SCHOLARONE™
Manuscripts

I, the Submitting Author has the right to grant and does grant on behalf of all authors of the Work (as defined in the below author licence), an exclusive licence and/or a non-exclusive licence for contributions from authors who are: i) UK Crown employees; ii) where BMJ has agreed a CC-BY licence shall apply, and/or iii) in accordance with the terms applicable for US Federal Government officers or employees acting as part of their official duties; on a worldwide, perpetual, irrevocable, royalty-free basis to BMJ Publishing Group Ltd ("BMJ") its licensees and where the relevant Journal is co-owned by BMJ to the co-owners of the Journal, to publish the Work in this journal and any other BMJ products and to exploit all rights, as set out in our licence.

The Submitting Author accepts and understands that any supply made under these terms is made by BMJ to the Submitting Author unless you are acting as an employee on behalf of your employer or a postgraduate student of an affiliated institution which is paying any applicable article publishing charge ("APC") for Open Access articles. Where the Submitting Author wishes to make the Work available on an Open Access basis (and intends to pay the relevant APC), the terms of reuse of such Open Access shall be governed by a Creative Commons licence – details of these licences and which Creative Commons licence will apply to this Work are set out in our licence referred to above.

Other than as permitted in any relevant BMJ Author's Self Archiving Policies, I confirm this Work has not been accepted for publication elsewhere, is not being considered for publication elsewhere and does not duplicate material already published. I confirm all authors consent to publication of this Work and authorise the granting of this licence.

Confidential: For Review Only

Health-related quality of life and economic burden of childhood pneumonia in China: a multi-region study

Junyang Gao [1,2*], BSc

Jingzhi Fan [1,2*], BSc

Huijun Zhou[3], PhD

Mark Jit[4], PhD

Pei Wang[1,2], PhD

[1] School of Public Health, Fudan University, Shanghai 200032, China

[2] Key Lab of Health Technology Assessment, National Health Commission of the People's Republic of China (Fudan University), Shanghai, China

[3] Business School, University of Shanghai for Science and Technology, Shanghai, China

[4] Department of Infectious Disease Epidemiology, Faculty of Epidemiology and Population Health, London School of Hygiene & Tropical Medicine, London, United Kingdom.

[*] The authors contribute equally

Corresponding to:

Pei Wang,

School of Public Health,

Fudan University

130 Dong An Road, Shanghai 200032, China.

E-mail: wang_p@fudan.edu.cn.

Text word count: 3557 words

Keywords: pneumonia, child, health-related quality of life, economic burden

Confidential: For Review Only

Abstract

Objective: To systematically investigate the health-related quality of life (HRQOL) and economic burden of children with pneumonia in different regions of China.

Study design: The study recruited a series of children under 5 years hospitalized for pneumonia in Shanghai, Zhengzhou, and Kunming from January to October 2019.

Health utility was assessed using the proxy version of EQ-5D-Y by interviewing patients' guardians face-to-face. The assessment was administered twice at patients' admission and discharge. Cost incurred for receiving the hospitalization were collected. Multiple linear regression and quantile regression were used to explore factors of EQ-5D-Y health utility score (HUS) and costs, respectively.

Results: A total of 501 pediatric patients with a median age (interquartile range, IQR) of 1.5 (0.83-2.71) years were included in the analysis. The mean HUS (standard deviations, SD) of the patients was 0.78 (0.18) at admission, and increased to 0.96 (0.10) at discharge. Some patients (14.2%) still felt worried, sad or unhappy after hospitalization. The mean hospitalization cost and total cost were RMB 5,859 (773 Euros) and RMB 6,439, respectively. The HUS was lower and the economic burden was heavier for the children in Zhengzhou. Apart from region, type of work, insurance status and hospital days were also related to the baseline HUS or HUS increment after treatment; insurance status, VAS score at discharge, guardians' employment and hospitalization days were associated with the costs.

Conclusion: The children with pneumonia have poor baseline HRQOL, and many of them still have mental health problems after treatment. The economic burden varied significantly across regions and is heavy for the patients' families in less developed areas (i.e., Zhengzhou and Kunming).

Keywords: pneumonia, child, health-related quality of life, economic burden

Confidential: For Review Only

Key message

What is already known: Many studies have found that children with pneumonia had poorer health-related quality of life (HRQOL), especially in social and psychological functioning dimensions; pneumonia also placed a heavy economic burden on the children's family.

What this study adds: The study provided health utility score (HUS) for Chinese children with pneumonia. The study also compared HRQOL and economic burden among different regions in China.

How this study might affect research, practice or policy: We found that many pediatric patients still had mental health problems after treatment, suggesting that their psychological status deserves more attention during and after clinical care. We also identified significant regional differences in HRQOL and economic burden, as well as other influencing factors.

Confidential: For Review Only

Introduction

Pneumonia is a significant type of lower respiratory tract infection affecting children worldwide(1), with serious impact on both physical and psychological aspects of health, such as coughing, breathlessness and irritability(2). It is the major single cause of death in children, causing approximately 1 million deaths annually, or 15% of an estimated 6.3 million deaths in children aged under 5 years(3). Studies have also shown that pneumonia is one of the top 5 causes of death in children under 5 years in China, accounting for 12.4% of deaths(4, 5). Pneumonia causes not only physical impairment, but also psychological, debilitating and social adjustment problems to the affected child. Hence, health-related quality of life (HRQOL), a comprehensive health outcome measure is required to holistically reflect the disease influence. Moreover, the HRQOL information could also be translated into health utility score (HUS) reflecting the value of HRQOL for use in economic evaluation if it is measured by utility instruments such as EQ-5D.

International and domestic studies have assessed HRQOL of pediatric patients with pneumonia using HRQOL instruments such as the Children's Quality of Life Scale (PQL 4.0)(6, 7) and the Generic Quality of Life Inventory-74 (GQOLI-74)(8). They found that the pediatric patients had poorer HRQOL, especially in social and psychological functioning dimensions. Studies in Indonesia and Thailand have also reported HUS of the patients using the proxy version of EQ-5D(9, 10). However, no studies have yet provided available data on HUS for the Chinese pediatric patients.

In China, some kinds of the costs incurred from pneumonia treatment, such as tests, anti-infective drugs, and bed charges, are not fully reimbursed according to Chinese healthcare system. Patients thus have to pay for the excess costs themselves. As a result, pneumonia also places a heavy financial burden on the children's family. Several studies have assessed its economic burden in childhood respiratory infections and pneumonia in China(11, 12). Wang et al. investigated the average length of stay (LOS) in hospital and hospitalization cost of 8,334 children with acute respiratory

Confidential: For Review Only

disease under 5 years in Gansu province from 2015 to 2018. They found that the LOS was 6.6 days and the expenditure was RMB 5,613 exceeding 30% of the per capita disposable income of the province in 2018. Similarly, Wang et al. conducted a retrospective study of 86 children with pneumonia under 5 years in a community in Shanghai during 2012. The study indicated the average total cost including outpatient cost, hospitalization cost, out-of-pocket drug cost, traffic cost, lost work cost was RMB 4,017, which accounted for 10% of the per capita disposable income of Shanghai in 2012. However, those studies were based on the patients in a single place, which may not be representative of the broader Chinese population and thus lead to limited extrapolation.

Hence, this study aimed to systematically investigate the HRQOL and economic burden of children with pneumonia from three different regions in China with divergent economic development levels.

Methods

Study design and patients

This study measured HRQOL of hospitalized pneumonia children at admission and discharge. Various costs associated with hospitalization were collected and aggregated. The study was conducted from January to October 2019 in three provincial capitals of sampled provinces representing different socio-economics statuses. The cities were Shanghai (eastern region), Zhengzhou (central region), and Kunming (western region), representing high, medium, and low status, respectively. In each city, a general or special (e.g., children's hospital) tertiary hospital was selected as the sampling hospital.

The inclusion criteria for the study were 1) younger than five years old, 2) clinical diagnosis of pneumonia, and 3) no concomitant diseases. All the pediatric patients hospitalized in the studying sites at the time of study were assessed for the eligibility by trained interviewers. Once a patient was eligible, his/her parents/guardians were invited to participate in the study.

Confidential: For Review Only

The consenting parents/guardians were interviewed face-to-face twice. The first interview was conducted within the first two days of patients' admission. A questionnaire was used to assess the child's HRQOL and to collect information on the child's (i.e., gender, age and medical insurance) and his/her guardian's sociodemographic characteristics (i.e., gender, age, education level, marriage status, employment, and monthly income level, race, domicile, religion, relationship with the child). The second interview was performed when the child was discharged from the hospital, and assessed the child's HRQOL again, as well as the total hospitalization costs including any household expenses, insurance payments (if any), lost work time, and the length of stay (LOS).

The study was supported by grants from the National Institute for Health Research (NIHR) (grant no. 16/137/109) using UK aid from the UK Government to support global health research. The views expressed in this publication are those of the author(s) and not necessarily those of the NIHR or the UK Department of Health and Social Care.

Health-related quality-of-life Measurement

The EQ-5D-Y is used as the HRQOL measure in the study. It is the youth version of the widely used EQ-5D that is more aligned with children's perceptions and understanding. It has five dimensions each with five response options: *walking about*, *looking after myself*, *doing usual activity*, *having pain or discomfort*, *feeling worried, sad or unhappy*.

The five response options of each dimension of Y-5L are *no problems* (level 1), *a little bit of problems* (level 2), *some problems* (level 3), *a lot of problems* (level 4), *extreme problems* (level 5)(13). As a result, it defines a total of 3,125 (5^5) health states by combing the responses of each dimension. A HUS can be assigned to each health state using a utility value set. Since the utility value set for the Y-5L is not available currently, the study used the Chinese value set of the EQ-5D-5L to calculate the HUS for the Y-5L health states(14). In addition, Y-5L uses a visual analog scale

Confidential: For Review Only

(VAS) with a score of 100 at the top for "the best imaginable health" and 0 at the bottom for "the worst imaginable health".

Given that the children aged 0-5 years were cared by their parents, it was assumed that the dimension “looking after myself” is irrelevant. For the children under 18 months who were unable to stand, the dimension “walking about” is assumed irrelevant(15, 16).

Economic Burden of Pneumonia

The economic burden of pneumonia was estimated by calculating direct medial cost (i.e., hospitalization cost including out-of-pocket cost, insurance payments if covered by insurance), non-medical cost (i.e., traffic cost), as well as indirect cost. Indirect cost was derived by estimating the loss of productivity of guardian using human capital approach. That is, the loss of work hour multiplies hourly wages based on annual average income in China in 2021 (8 hour per work day, and 250 work days per year). All cost data was adjusted to 2021 Chinese CNY using the latest published CPI index in China.

Statistical analysis

Descriptive statistical analyses were adopted to depict sociodemographic characteristics of the patients and their guardians, distributions of Y-5L dimension responses, Y-5L HUS and VAS score, as well as economic burden data. Categorical variables were presented using number and percentages; continuous variables were described using mean, standard deviations (SD) and range; and age is described using median and interquartile range (IQR). Chi-square test, median test or ANOVA test were used to compare the characteristics of the patients/guardians, HRQOL and economic burden of the patient among the three cities, and HRQOL between admission and discharge whenever appropriate. Comparison of HUS, VAS score and cost using the Tukey post hoc test(17) between samples were further conducted to determine which pairwise groups were different.

https://mc.manuscriptcentral.com/bmjpo

Confidential: For Review Only

Multiple linear regression was used to analyze the factors influencing the HUS of children with pneumonia. HUS at baseline and its difference between baseline and discharge were used as the two dependent variables. According to previous studies(18, 19), hospitalization days and demographic characteristics including region, gender, age, insurance status of the children; and education level, employment of the guardians may have a significant effect on HUS. These factors were adopted as the independent variables in the two models (hospital days was adopted only in the model for HUS difference).

For the economic burden, quantile regression was used to analyze the impact factors. Referring to the previous studies (20, 21), three quantile points of 10%, 50% and 90% of hospitalization cost and total cost (hospitalization cost, transportation cost, and cost for productivity loss) were selected separately for the quantile regression analysis. The two costs were log-transformed to approximate normal distributions as the two dependent variables. Based on the evidence of prior studies(11, 12, 22, 23), gender, age, hospital days, insurance status, and disease prognosis may have a significant impact on costs and adopted as independent variables in the two models. Also, VAS score reflecting the disease prognosis, region and employment of guardians were also adopted in the models.

SPSS23.0 and SPSS 26.0 was used for all the analysis, and the threshold for significant differences was a P-value less than 0.05.

Results

Characteristics of study subjects

Table 1 shows the sociodemographic characteristics of children with pneumonia and their guardians. A total of 501 patients were included in analysis: 173, 120 and 208 in Shanghai, Zhengzhou and Kunming, respectively. The median age (IQR) of the patients was 1.50 (0.83-2.71) years, and the proportions of boys and girls were close (51.5% vs. 48.5%). A relatively high percentage of the children were without

Confidential: For Review Only

medical insurance (48.5%). More than half of the guardians were female (53.9%) with a median age (IQR) of 31.00 (28.00-35.00) years, and the majority of them were parents of the children (91.6%). They were mostly Han Chinese (90.6%), non-religious (96.8%), married (98.0%), from urban area (67.5%), university-educated or above (42.5%), enterprise/self-employed (41.9%). Most of them (57.8%) had family monthly income over 10,000 CNY (1319 Euros). The characteristics of the patients and their guardians differed among the three regions except for marital status (P=0.25).

Table 1 Sociodemographic characteristics of children with pneumonia and their guardians among the three regions

Variables	Overall N=501	Shanghai N=173	Zhengzhou N=120	Kunming N=208	P-value[a]
**Characteristics of the child**					
**Gender**					< 0.01
Boys	258 (51.5)	72 (41.6)	63 (52.5)	123 (59.1)	
Girls	243 (48.5)	101 (58.4)	57 (47.5)	85 (40.9)	
**Age(year)**					< 0.01
Median	1.50	1.00	2.50	1.17	
IQR	0.83-2.71	0.92-2.00	2.08-3.50	0.40-2.33	
**Medical Insurance**					< 0.01
Yes	185 (36.9)	91 (52.6)	41 (34.2)	53 (25.5)	
None	243 (48.5)	80 (46.2)	79 (65.8)	84 (40.4)	
Unknown	71 (14.2)	-	-	71 (34.1)	
**Guardianship characteristics**					
**Age(year)**					0.04
Mean (SD)	31.00	31.00	32.50	31.00	
Range	28.00-35.00	28.00-37.00	30.00-35.00	27.00-34.00	
**Gender**					< 0.01
Male	209 (41.7)	57 (32.9)	42 (35.0)	110 (52.9)	
Female	292 (58.3)	116 (67.1)	78 (65.0)	98 (47.1)	
**Relationship**					< 0.01
Father	189 (37.7)	35 (20.2)	42 (35.0)	112 (53.8)	
Mother	270 (53.9)	105 (60.7)	78 (65.0)	87 (41.8)	
Other[b]	42 (8.4)	33 (19.1)	-	9 (4.3)	
**Ethnic**					< 0.01
Han	454 (90.6)	171 (98.8)	118 (98.3)	165 (79.3)	
Others	47 (9.4)	2 (1.2)	2 (1.7)	43 (20.7)	
**Religion**					0.01

Confidential: For Review Only

None	485 (96.8)	170 (98.3)	118 (98.3)	197 (94.7)	
Yes or refused to answer	13 (2.6)	3 (1.7)	2 (1.6)	11 (5.3)	
**Marriage Status**					0.25
Married	491 (98.0)	170 (98.3)	120 (100.0)	201 (96.6)	
Singles and others	10 (2.0)	3 (1.8)	-	7 (3.4)	
**Domicile**					< 0.01
City	241 (48.1)	102 (59.0)	95 (79.2)	44 (21.2)	
Town	97 (19.4)	43 (24.9)	20 (16.7)	34 (16.3)	
Rural	161 (32.1)	27 (15.6)	5 (4.2)	129 (62.0)	
**Education**					< 0.01
Elementary school and below	28 (5.6)	11 (6.3)	-	17 (8.2)	
Junior High School	104 (20.8)	14 (8.1)	2 (1.7)	88 (42.3)	
High School	88 (17.6)	30 (17.3)	15 (12.5)	43 (20.7)	
College	68 (13.6)	23 (13.3)	25 (20.8)	20 (9.6)	
University and above	213 (42.5)	95 (54.9)	78 (75.0)	40 (19.2)	
**Employment**					< 0.01
Enterprise/self-employed	210 (41.9)	77 (44.5)	83 (69.2)	50 (24.0)	
Civil servants / Public Institution	83 (16.6)	20 (11.6)	24 (20.0)	39 (18.8)	
Full-time home/housewife	73 (14.6)	37 (21.4)	5 (4.2)	31 (14.9)	
Retirement	21 (4.2)	18 (10.4)	-	3 (1.4)	
Farming	50 (10.0)	11 (6.4)	3 (2.5)	36 (17.3)	
Temporary workers	39 (7.8)	1 (0.6)	4 (3.3)	34 (16.3)	
Unemployment	21 (4.2)	7 (4.1)	1 (0.8)	13 (6.2)	
Other or refused to answer	3 (0.6)	1 (0.6)	-	2 (1.0)	
**Family monthly income (CNY)**					< 0.01
<5000 (660Euros)	83 (16.6)	-	3 (2.5)	80 (38.5)	
5000-10000	97 (19.4)	-	20 (16.7)	77 (37.0)	
10000-20000	76 (15.2)	9 (5.2)	47 (39.2)	20 (9.6)	
20,000-30,000	75 (15.0)	37 (21.4)	34 (28.3)	4 (1.9)	
> 30,000	117 (23.4)	94 (54.3)	16 (13.3)	7 (3.3)	
Unknown or refused to answer	52 (10.4)	32 (18.5)	-	20 (9.6)	

IQR: interquartile range; SD: standard deviations; CNY: Chinese Yuan.

[a]Comparison of population characteristics among the three regions. Categorical variables and continuous variables were analyzed using chi-square test and ANOVA test, respectively.

[b]This refers to a guardian such as a non-parental relative of the affected child.

Health-related quality-of-life of the children

Among the 501 patients, 394 patients responded to the EQ-5D-Y scale at admission, and 372 patients responded again at discharge. The percentage of reporting problems in the Y-5L dimensions at admission and discharge is shown in online supplemental file 1. At admission, the children had more problems in *having pain or*

Confidential: For Review Only

discomfort (85.5%) and *feeling sad, worried, or unhappy* (83.0%) dimensions; and the children in Zhengzhou had the highest prevalence of problems in each dimension. At discharge, the children's health status was significantly improved and no cases had very severe problems (level 5) in all the dimensions, while a relatively high proportion of problems was still observed in *feeling worried, sad or unhappy* dimension (19.0%). Similarly, the children in Zhengzhou tended to have more problems.

Table 2 reports the Y-5L HUS and VAS score of children with pneumonia in the three locations. The mean HUS of the patients at admission was 0.78, and the patients in Shanghai and Zhengzhou reported the highest (0.84) and lowest HUS (0.66), respectively. The HUS at discharge was significantly increased with the mean value being 0.96, and the children from Kunming and Zhengzhou had higher (0.99) or lower (0.90) HUS. The results of multiple comparisons showed significant difference in HUS between Shanghai and Zhengzhou, Kunming and Zhengzhou at both admission and discharge, while the difference between Shanghai and Kunming was insignificant. The difference in HUS at admission and discharge for children in Zhengzhou was significantly greater than the difference in the other two regions. The mean VAS score of children in the three sites at both admission and discharge was also significantly different (62.9 vs. 89.2, $P<0.05$), with the children in Kunming having the highest score at both admission (65.4) and discharge (91.9), those in Zhengzhou and Shanghai having the lowest score at admission (58.3) and at discharge (77.2), respectively

Confidential: For Review Only

Table 2 EQ-5D-Y health utility and EQ-VAS scores of children with pneumonia in the three locations

	Total<br>Admission: N=394<br>Discharge: N=372	Shanghai (1)<br>Admission: N=66<br>Discharge: N=44	Zhengzhou (2)<br>Admission: N=120<br>Discharge: N=120	Kunming (3)<br>Admission: N=208<br>Discharge: N=208	Post hoc analysis[a]	P-value[b]
**Utility admission**						
Mean (SD)	0.78 (0.18)	0.84 (0.07)	0.66 (0.21)	0.82 (0.15)	(1) > (2); (3) >	<0.001
Range	-0.04-1.00	0.63-1.00	-0.04-0.95	0.25-1.00	(2)	
**VAS admission**						
Mean (SD)	62.9（16.6）	63.5（14.2）	58.3（15.5）	65.4（17.4）	（3）>（2）	<0.01
Range	15–100	40–95	15–84	20–100		
**Utility discharge**						
Mean (SD)	0.96（0.10）	0.97（0.04）	0.90（0.15）	0.99（0.04）	（1）>（2）；（3）>	<0.001
Range	0.27–1.00	0.85–1.00	0.27–1.00	0.67–1.00	（2）	
**VAS discharge**						
Mean (SD)	89.2（13.0）	77.2（9.5）	88.9（10.7）	91.9（13.5）	（2）>（1）；（3）>	<0.01
Range	40–100	60–95	47–100	40–100	（1）	
**Utility difference**						
Mean (SD)	0.19（0.16）	0.15（0.06）	0.24（0.21）	0.17（0.14）	（1）<（2）；（2）>	<0.001
Range	–0.05–1.04	0.04–0.32	0–1.04	–0.05–0.75	（3）	
**VAS difference**						
Mean (SD)	27.1（13.5）	20.1（7.7）	30.6（14.3）	26.5（13.4）	（2）>（1）；（2）>	<0.01
Range	0–74	5–40	10–74	0–60	（3）	

SD, standard deviations.

[a]Comparison using the Tukey post hoc test.

[b]Calculated using ANOVA test.

Confidential: For Review Only

Economic Burden of Pneumonia

The economic burden manifested by hospitalization cost and total cost summing of hospitalization cost, transportation cost, and indirect cost are shown in Table 3. The mean hospitalization cost of the three regions was RMB 5,859 and the children in Zhengzhou (RMB 8,667) had much higher cost than that in Shanghai (RMB 3,100) and Kunming (RMB 6,522). The mean transportation cost was RMB 110, and the children in Kunming (RMB 220) had the highest cost followed by the children in Shanghai (RMB 4) and Zhengzhou (RMB 73). The average hospitalization days was 8.0 days: the days were 10.6 days, 8.2 days, and 4.8 days for the children in Kunming, Shanghai, and Zhengzhou, respectively. Correspondingly, the average time of loss work of guardians in Kunming (65.9 hours) was substantially higher than that in Shanghai (6.5 hours) and Zhengzhou (33.1 hours). This also resulted in the highest indirect cost in Kunming (RMB 710), followed by Zhengzhou (RMB 444), and Shanghai (RMB 187). For the total cost, the average amount was RMB 6,439, and the patients in Zhengzhou had the highest amount (RMB 9,183), followed by Kunming (RMB 7,464), and Shanghai (RMB 3,293).

Table 3 Economic burden of disease in children with pneumonia

	**Overall** N=501	**Shanghai** (1)	**Zhengzhou** (2)	**Kunming** (3)	**Post hoc comparison**[a]	**P value**[b]
**Traffic cost**						<0.01
Mean (SD)	110. 0（270. 1）	4. 3（9. 8）	72. 9（138. 2）	219. 9（378. 6）	(1) < (3);	
Range	0–3514	0–60	0–780	0–3514	(2) < (3)	
**Out-of-pocket cost**						
Mean (SD)	3816. 1(3981. 9)	2036. 6（1077. 7）	3813. 8(1990. 3)	5229. 1（5473. 3）	(1) < (2) < (3)	<0. 01
Range	471–60420	471–8236	1350–10416	504–60420		
**Hospitalization cost**					(1) < (2);	
Mean (SD)	5859. 2（5486. 5）	3099. 7（1474. 2）	8666. 6(3965. 2)	6521. 5（7098. 9）	(1) < (3);	<0. 01
Range	302–80560	987–8261	3114–18998	302–80560	(2) > (3)	
**Loss of work time (hour)**						<0. 01
Mean (SD)	37. 9（81. 1）	6. 5（3. 9）	33. 1（15. 8）	65. 9（121. 0）		
Range	0–1200	0–10	9–80	0–1200	–	
**Indirect cost**[c]						
Mean (SD)	465. 79（967. 89）	187. 40（171. 73）	443. 50 (215. 60)	710. 20（1444. 48）	(1) < (3);	<0. 01
Range	0–15399. 60	0–390. 14	107. 24–1072. 44	0–15399. 60	(2) < (3)	
**Hospitalization days**						<0. 01

Confidential: For Review Only

Mean (SD)	8.0（6.6）	4.8（1.9）	8.2（3.0）	10.6(9.1）	–	
Range	0–50	1–11	5–15	0–50		
**Total Cost[d]**						<0.01
Mean (SD)	6439.37（5772.99）	3292.52（1511.23）	9183.00（4085.72）	7463.64（7437.87）	（1）<（3）<（2）	
Range	918.09–80560.48	986.86–8701.53	3370.06–19346.78	918.09–80560.48		

SD, standard deviations.

[a]Comparison using the Tukey post hoc test.

[b]Calculated using ANOVA test.

[c]Indirect cost: loss of work hour multiples income per hour that converted from annual average income in China 2021 (8 hour per work day, and 250 work days per year).

[d]Total cost includes hospitalization cost, traffic cost and indirect cost.

Regression analyses

Table 4 present the significant coefficients generated from the multiple linear regressions. Region, insurance status and type of work were significantly associated with baseline HUS (Table 4). That is, the children in Zhengzhou, without insurance, and their guardians who were temporary workers had lower baseline HUS. Region and hospitalization days were significantly correlated with the difference in HUS: the children in Zhengzhou and with longer hospital days were more likely to have higher difference in HUS (Table 4).

Table 4 Multiple regression analysis of baseline health utility score and health utility score difference between baseline and follow-up

Variables	Baseline HUS		HUS difference (discharge-admission)	
	Coefficient (95% CI)[a]	P-value[a]	Coefficient (95% CI)[a]	P-value[a]
**Region (Shanghai[b])**				
Zhengzhou	-0.17（-0.22, -0.12）	<0.01	0.06（0.01, 0.12）	0.02
Kunming	0.04（-0.01, 0.1）	0.12	-0.06（-0.12, 0）	0.06
**Gender**	-0.02（-0.05, 0.02）	0.32	0.01（-0.02, 0.04）	0.48
**Age**	0.002（-0.01, 0.02）	0.72	-0.003 (-0.02, 0.01)	0.63
**Insurance status**	-0.001（-0.001, 0）	0.01	0.001（0, 0.001）	0.06
**Education**				
**（Elementary school and below[b]）**				
Junior High School	-0.001（-0.09, 0.09）	0.98	0.00（-0.09, 0.09）	1.00
High School	-0.03（-0.09, 0.03）	0.29	0.02 (-0.05, 0.08）	0.64
College	-0.03（-0.09, 0.02）	0.20	0.04（-0.02, 0.09）	0.16
University and above	0.02（-0.03, 0.07）	0.43	-0.04（-0.09, 0.01）	0.11
**Employment Status**				

Confidential: For Review Only

**(Enterprise/self-employed[b])**				
Civil servants / Public Institution	-0.02 (-0.07, 0.02)	0.30	0.03 (-0.02, 0.07)	0.21
Full-time home/housewife	0.03 (-0.03, 0.09)	0.39	-0.03 (-0.09, 0.03)	0.33
Retirement	0.06 (-0.05, 0.16)	0.27	-0.03 (-0.15, 0.09)	0.60
Farming	-0.03 (-0.09, 0.03)	0.32	0.02 (-0.04, 0.08)	0.50
Temporary workers	-0.08 (-0.14, -0.02)	0.01	0.05 (-0.01, 0.11)	0.08
Unemployment	-0.07 (-0.15, 0.02)	0.13	0.04 (-0.05, 0.13)	0.37
**Hospitalization days**	-	-	0.01 (0.002, 0.01)	<0.01

Abbreviations: HUS, health utility score; CI, confidence interval.

[a]Calculated using multiple linear regression.

[b]Reference category.

Confidential: For Review Only

The results of quantile regression analysis showed that region, insurance status, hospital days, VAS score at discharge and employment of guardians were significantly associated with both hospitalization and total costs (Table 5). Specifically, region had a significant effect at almost all quartiles, and the children in Zhengzhou and Kunming had higher costs. In addition, the degree of the effect was higher at the lower quartile than at the higher quartile for children in Zhengzhou. The two costs were significantly lower for the children without insurance and with shorter hospitalization days at the middle and high quartiles, and the effects of hospitalization days were stronger in the higher quartile. For children with higher VAS score at discharge, the two costs were significantly higher at the low and middle quantiles. At the low quantile, the two costs were significantly lower for children whose guardians were civil servant or public institution worker, and total cost was also lower for the children with retired working guardians. For children with guardians who were temporary workers, the two costs were significantly lower at almost all quartiles.

Table 5 Quantile regression analysis of total cost and hospitalization cost

**Variables**	**Hospitalization cost**			**Total Cost[a]**		
	q=0.1	q=0.5	q=0.9	q=0.1	q=0.5	q=0.9
**Region (Shanghai[b])**						
Zhengzhou	1.06**	0.91**	0.71**	0.96**	0.86**	0.74**
Kunming	0.35	0.56**	0.53**	0.62**	0.64**	0.66**
**Gender**	-0.03	-0.07	-0.01	-0.03	-0.07	-0.01
**Age**	-0.03	0.002	-0.01	0.002	0.01	-0.002
**Insurance status**	0.003	-0.003*	-0.004*	0.001	-0.004**	-0.004**
**VAS-discharge**	0.02**	0.01*	0.001	0.01**	0.01*	0.000
**Employment Status**						

**(Enterprise/self-employed[b])**						
Civil servants / Public Institution	-0.31*	-0.07	-0.04	-0.26**	-0.08	-0.04
Full-time home/housewife	-0.04	0.08	0.07	-0.12	0.03	0.04
Retirement	-0.40	-0.22	0.14	-0.56*	-0.36	0.04
Farming	-0.13	-0.05	-0.11	-0.03	-0.05	0.04
Temporary workers	-0.54*	-0.23*	-0.26*	-0.32*	-0.16	-0.27*
Unemployment	0.13	0.07	0.13	-0.08	0.05	0.03
**Hospitalization days**	0.01	0.02**	0.08**	0.01	0.02**	0.07**

[a]Total cost includes hospitalization cost, traffic cost and indirect cost.

[b]Reference category.

***:P<0.05, **:P<0.01**

Discussion

We found that the children with pneumonia had inferior health status both physically and psychologically; and the disease economic burden varied greatly among regions. Moreover, we also identified several important factors influencing the HUS and economic burden. Therefore, this study deepens the understanding of HRQOL and economic burden of the children in China.

At admission, the majority of children had health problems especially in *having pain or discomfort*, *feeling worried*, *sad or unhappy* dimensions as well as low HUS and VAS score. Previous studies have also reported similar findings that the pediatric patients with pneumonia had poor HRQOL at admission, especially in social and psychological functioning dimensions(6, 7). When pneumonia occurred, the children had symptoms such as fever, cough, sore throat and breathlessness(24), which may bring physical discomfort to the children, affect their emotions and lead to psychological problems. We further found that although their health improved a lot at discharge, many of them were still feeling worried, sad, or unhappy at discharge. Thus, their psychological condition should deserve more attention in the post-discharge period.

Among the three regions, the children in Zhengzhou had worse baseline HUS, which was probably due to that they were mainly from urban area containing more risk factors that may contribute to the development of pneumonia in childhood(25). The children without insurance also reported poorer baseline HUS. Some studies revealed that individuals who

Confidential: For Review Only

have health insurance reported a higher quality of life than those without health insurance(26, 27). In addition, the children also had poorer baseline HUS when their guardians were temporary workers. An Israeli study showed that both parental unemployment and workday loss affected the HRQOL of children with pneumonia as well(28). Temporary work means unstable income, making it difficult to provide good care to the children. The children in Zhengzhou had larger HUS difference. Since the patients were discharged from hospitals because their health was recovered and their HUS at discharge was close to full health, the identified association could be attributed to the HUS at baseline. That is, the children in Zhengzhou had worse baseline HUS. In addition, children with longer hospital days had higher HUS difference, probably because longer treatment time corresponds to better treatment effect.

We found that the average hospitalization and total costs were comparable to the previous results for the Chinese children under 5 years with pneumonia(11, 12): the average hospitalization cost was RMB 5,771 in Gansu and the average total cost was RMB 4,642 in Shanghai after being adjusted for 2021 CNY. The costs were also similar to the costs of other common childhood infectious diseases in China. According to 2021 CNY, the average hospitalization costs for children in Shenyang with scarlet fever and hand, foot and mouth disease were RMB 4,979 and RMB 5,050, respectively(29); the average total cost for inpatient cases with chickenpox in school-age children was RMB 3,312 in Shenzhen(30). On the other hand, we identified the disease economic burden varied greatly among the regions: the total cost was calculated to be 1.9% of the local per capita GDP in Shanghai, 9.6% in Zhengzhou, and 8.7% in Kunming, respectively. The finding indicated that the disease places severer economic burden on the children's families in less economically developed areas. The government thus could take necessary activities (e.g., increasing the reimbursement rate of medical insurance) to alleviate the burden of the families.

The children in Zhengzhou had higher hospitalization cost. This may be due to their worse baseline health status, requiring more medical resources and thus higher costs. For the transportation and indirect costs, the patients in Kunming had higher amount. Kunming is the capital city of Yunnan province, which had a relatively low level of socio-economic

Confidential: For Review Only

development and a lack of medical resources compared to other regions of China. Hence, the patients in other cities of the province have to concentrate in Kunming, leading to the higher costs.

The children without medical insurance had significantly lower hospitalization and total costs. A previous study had indicated that relying solely on out-of-pocket payment provided obstacles for treatment access, skewing treatment seeking towards those affordable(23), leading to lower costs. The two costs were also significantly associated with hospitalization days, and similar findings had been reported in a previous study that shortening the number of ineffective hospitalization days helped to reduce the financial burden(22). In the study, we found that children with higher VAS score at discharge had higher hospitalization and total costs. This may be because achieving better health required more consumption of medical services. We also found that the two costs were significantly higher for the children whose guardians were enterprise or self-employed workers. This was probably because they may have better economic conditions and were willing to spend more for their children to get better treatment.

This study has several limitations which should be noted. First, the study was conducted in three areas in China, which may not be representative of the entire country or other regions with different socioeconomic statuses. Also, only pediatric patients who were hospitalized at the time of the study were included; while those who were managed on an outpatient basis or treated at primary care clinics were not included. In addition, there were missing values in the responses to the EQ-5D-Y dimensions for the patients in Shanghai. These may have limited the representativeness of the study population and affected the generalizability of our findings. Second, due to the study design and resources, we did not enroll a control group of children without pneumonia for comparison. Thus, it may be difficult to attribute the observed results exclusively to pneumonia. Third, the Y-5L was designed mainly for children over 6 years of age, and the reliability and validity of its proxy version need to be further tested when it is applied to the guardians of children under 5 years. Also, the Chinese Y-5L value set has not been available, so the Chinese EQ-5D-5L value set was used to calculate the HUS instead. This introduces some uncertainty in the accuracy of the HRQOL measurements.

Confidential: For Review Only

Fourth, we did not collect certain clinical characteristics including disease severity and treatment modality, thus their influence on HRQOL and economic burden cannot be assessed. Fifth, although we excluded the pneumonia patients with other acute diseases, there may be undiagnosed comorbidities during data collection, which may affect HRQOL scores. Last but not least, the study lacked long-term follow-up, thus it was not possible to know the trajectory of HRQOL and economic burden over time.

Conclusion

The pediatric patients with pneumonia mainly suffered from pain, discomfort and felt sad, worried, or unhappy at admission, and many of them still had mental health problems after treatment. Region, insurance status, hospitalization days and employment of guardians were the four factors influencing their HRQOL. The economic burden of the disease varied significantly across regions and was heavy for the patients' families in less developed areas. Insurance status of the patients, employment status of their guardians, VAS score at discharge and hospitalization days were also associated with the burden.

Acknowledgements: We gratefully acknowledge the study participants and their families. We thank the hospitals for their support and cooperation with our data collection.

Contributors: JYG: Methodology, data analysis, manuscript writing (original draft). JZF: Data analysis, manuscript writing (original draft and revision). PW: Conceptualization, methodology, critical manuscript revision, supervision and project administration. All authors critically revised the manuscript and approved the final version of manuscript.

Funding: This research was funded by the National Institute for Health Research (NIHR) (GHR 16/137/09) using UK aid from the UK Government to support global health research. The views expressed in this publication are those of the author(s) and not necessarily those of the NIHR or the UK government.

Confidential: For Review Only

Ethic: This study was conducted according to the guidelines of the Declaration of Helsinki and approved by the Institutional Review Board of School of Public Health, Fudan University (IRB00002408).

Competing interests: The authors declare no competing interests

Patient and public involvement: Patients and/or the public were not involved in the design, conduct, reporting or dissemination plans of this research

Provenance and peer review: Not commissioned; externally peer reviewed.

Data availability: The datasets analyzed during the current study are available from the corresponding author on reasonable request

Confidential: For Review Only

References

1. Lu Y-Y, Luo R, Fu Z. Pathogen distribution and bacterial resistance in children with severe community-acquired pneumonia. Zhongguo dang dai er ke za zhi = Chinese journal of contemporary pediatrics. 2017;19(9):983-8.

2. K W, P G, R P, A T, D M, A H. - Clinical symptoms and signs for the diagnosis of Mycoplasma pneumoniae in. D - 100909747. (- 1469-493X (Electronic)):- CD009175.

3. HJ Z, W B, L M, MP N. - Childhood pneumonia - the Drakenstein Child Health Study. D - 0404520. (- 0256-9574 (Print)):- 642-3.

4. Feng J, Yuan X, Zhu J, Li X, Miao L, He C, et al. Under-5-mortality rate and causes of death in China, 2000 to 2010. Chinese Journal of Epidemiology. 2012;33(6):558-61.

5. Song P, Theodoratou E, Li X, Liu L, Chu Y, Black RE, et al. Causes of death in children younger than five years in China in 2015: an updated analysis. Journal of Global Health. 2016;6(2).

6. Johnson J, Wilson KM, Zhou C, Johnson DP, Kenyon CC, Tieder JS, et al. Home Smoke Exposure and Health-Related Quality of Life in Children with Acute Respiratory Illness. Journal of Hospital Medicine. 2019;14(4):212-7.

7. Ma Z. Application effects of clinical nursing pathway in children with severe pneumonia. Medical Journal of Chinese People’s Health. 2020;32(24):150-1+4.

8. Niu D, Chen L, Li J. Application of safety warning nursing mode in the whole course of nursing for children with severe pneumonia. Journal of Clinical Psychosomatic Diseases. 2020;26(01):186-9.

9. Hadning I, Andayani TM, Endarti D, Triasih R. Health-Related Quality of Life Among Children With Pneumonia in Indonesia Using the EuroQoL Descriptive System Value Set for Indonesia. Value Health Reg Issues. 2021;24:12-6.

Confidential: For Review Only

10. Kulpeng W, Sornsrivichai V, Chongsuvivatwong V, Rattanavipapong W, Leelahavarong P, Cairns J, et al. Variation of health-related quality of life assessed by caregivers and patients affected by severe childhood infections. BMC Pediatr. 2013;13:122.

11. Wang S, Zhang T, Zhang X, Li H, Shi Y, Wang Y. Study on the prevalence characteristics and economic burden of acute respiratory diseases in children under 5 years old in Gansu. Maternal and Child Health Care of China. 2020;35(14):2548-52.

12. Wang J, Huang Z, Hu J, Jiang M, Lu J, Xu z, et al. Retrospective study on incidence and diagnosis of pneumonia in children under 5 years old in a community of Shanghai. Shanghai Journal of Preventive Medicine. 2018;30(7):552-6.

13. Zhou W, Shen A, Yang Z, Wang P, Wu B, Herdman M, et al. Patient-caregiver agreement and test-retest reliability of the EQ-5D-Y-3L and EQ-5D-Y-5L in paediatric patients with haematological malignancies. European Journal of Health Economics. 2021;22(7):1103-13.

14. Pan CW, Zhang RY, Luo N, He JY, Liu RJ, Ying XH, et al. How the EQ-5D utilities are derived matters in Chinese diabetes patients: a comparison based on different EQ-5D scoring functions for China. Qual Life Res. 2020;29(11):3087-94.

15. Zheng Y, Jit M, Wu JT, Yang J, Leung K, Liao Q, et al. Economic costs and health-related quality of life for hand, foot and mouth disease (HFMD) patients in China. Plos One. 2017;12(9).

16. JT W, M J, Y Z, K L, W X, J Y, et al. - Routine Pediatric Enterovirus 71 Vaccination in China: a Cost-Effectiveness. D - 101231360. (- 1549-1676 (Electronic)):- e1001975.

17. Jiao F, Wong CKH, Gangwani R, Tan KCB, Tang SCW, Lam CLK. Health-related quality of life and health preference of Chinese patients with diabetes mellitus managed in primary care and secondary care setting: decrements associated with individual complication and number of

Confidential: For Review Only

complications. Health Qual Life Outcomes. 2017;15(1):125.

18. MR T, R M-S, C Z, DR W, EE H, DP J, et al. - Obesity and Health-Related Quality of Life in Children Hospitalized for Acute. D - 101585349. (- 2154-1671 (Electronic)):- 841-8.

19. N M, GP P, SR H, T R, A U, MA P, et al. - Parents' Working Conditions in the Early COVID-19 Pandemic and Children's. D - 101304551. (- 1661-8564 (Electronic)):- 1605036.

20. MA O, F T, AE W, KB N, DK W, VJ F, et al. - Use of Quantile Regression to Determine the Impact on Total Health Care Costs of. D - 0372354. (- 1528-1140 (Electronic)):- 331-9.

21. Lei W, Yaya Y, Yini Z, Feng T, Xiaobin H. Analysis of influencing factors of hospitalizat ion expenses in elderly patients with injury based on quantile regress ion and random forest. Chinese Journal of Prevention and Control of Chronic Diseases. 2023;31(04):246-50.

22. Hu J, Xu B, Shen K. Hospitalization Cost and Influencing Factors of Pneumonia with Children in a Certain Hospital in Beijing. Chinese Journal of Health Informatics and Management. 2018;15(2):211-6.

23. Adamu AL, Karia B, Bello MM, Jahun MG, Gambo S, Ojal J, et al. The cost of illness for childhood clinical pneumonia and invasive pneumococcal disease in Nigeria. Bmj Global Health. 2022;7(1).

24. Liang W, Liu Y, Ren J. Observation on Efficacy of Cefixime Granules Combined with Ambroxol Oral Liquid in Treatment of Outpatient Pediatric Pneumonia. Evaluation and Analysis of Drug-Use in Hospitals of China. 2019;19(02):192-4.

25. Zhuge Y, Qian H, Zheng X, Huang C, Zhang Y, Zhang M, et al. Residential risk factors for childhood pneumonia: A cross-sectional study in eight cities of China. Environ Int. 2018;116:83-91.

26. M B, Thomas J r. - Health insurance coverage and health-related quality of life: analysis of 2000.

Confidential: For Review Only

D - 9103800. (- 1049-2089 (Print)):- 643-54.

27. RE G, L R, EG E, J D, L C. - Quality of life and associated characteristics in a large national sample of. D - 7805975. (- 0149-5992 (Print)):- 562-7.

28. Shoham Y, Dagan R, Givon-Lavi N, Liss Z, Shagan T, Zamir O, et al. Community-acquired pneumonia in children: quantifying the burden on patients and their families including decrease in quality of life. Pediatrics. 2005;115(5):1213-9.

29. Peng X. Hospitalization Cost Analysis of Common Infectious Diseases in Children from the Perspective of DRG [硕士]: China Medical University; 2022.

30. Wang T, Zheng Q, Wu Y, Yao Y, Kuang C, Li X. Investigation of economic burden caused by varicella in school aged children in Shenzhen. Disease Surveillance. 2021;36(10):1092-5.

Confidential: For Review Only

Guardians' responses to the EQ-5D-Y dimensions at admission and discharge

**Dimension**	**Overall**		**Shanghai**		**Zhengzhou**		**Kunming**		**P-value[a]**		
	Admission N=394	Discharge N=372	Admission N=66	Discharge N=44	Admission N=120	Discharge N=120	Admission N=208	Discharge N=208	Admission	Discharge	Comparison[b]
**Walking about**									<0.01	0.06	<0.01
No problems	152 (38.6%)	202 (54.3%)	59 (89.4%)	44 (100.0%)	27 (22.5%)	66 (55.0%)	66 (31.7%)	92 (44.2%)			
A little bit of problems	86 (21.8%)	32 (8.6%)	5 (7.6%)	0	56 (46.7%)	27 (22.5%)	25 (12.0%)	5 (2.4%)			
Some problems	16 (4.1%)	6 (1.6%)	0	0	10 (8.3%)	6 (5.0%)	6 (2.9%)	0			
A lot of problems	7 (1.8%)	2 (0.5%)	0	0	6 (5.0%)	1 (0.8%)	1 (0.5%)	1 (0.5%)			
Extreme problems	1 (0.3%)	0	0	0	1 (0.8%)	0	0	0			
＜18month[c]	132（33.5%）	130（34.9%）	2 (3.0%)	0	20 (16.7%)	20 (16.7%)	110 (52.9%)	110 (52.9%)			
**Doing usual activities**									<0.01	<0.01	<0.01
No problems	146 (37.1%)	319 (85.8%)	36 (54.5%)	43 (97.7%)	20 (16.7%)	77 (64.2)	90 (43.3%)	199 (95.7%)			
A little bit of problems	185 (47.0%)	45 (12.1%)	30 (45.5%)	1 (2.3%)	67 (55.8%)	35 (29.2)	88 (42.3%)	9 (4.3%)			
Some problems	46 (11.7%)	7 (1.9%)	0	0	23 (19.2%)	7 (5.8)	23 (11.1%)	0			
A lot of problems	11 (2.8%)	1 (0.3%)	0	0	7 (5.8%)	1 (0.8)	4 (1.9%)	0			
Extreme problems	6 (1.5%)	0	0	0	3 (2.5%)	0	3 (1.4%)	0			
**Having pain or discomfort**									<0.01	<0.01	<0.01
No problems	56 (14.2%)	318 (85.5%)	6 (9.1%)	40 (90.9%)	4 (3.3%)	79 (65.8%)	46 (22.1%)	199 (95.7%)			
A little bit of problems	223 (56.6%)	43 (11.6%)	54 (81.8%)	4 (9.1%)	60 (50.0%)	30 (25.0%)	109 (52.4%)	9 (4.3%)			
Some problems	93 (23.6%)	9 (2.4%)	6 (9.1%)	0	42 (35.0%)	9 (7.5%)	45 (21.6%)	0			
A lot of problems	20 (5.1%)	2 (0.5%)	0	0	12 (10.0%)	2 (1.7%)	8 (3.8%)	0			
Extreme problems	2 (0.5%)	0	0	0	2 (1.7%)	0	0	0			
**Feeling worried, sad or unhappy**									<0.01	<0.01	<0.01
No problems	67 (17.0%)	301 (81.0%)	8 (12.1%)	22 (50.0%)	1 (0.8%)	78 (65.0%)	58 (27.9%)	201 (96.6%)			
A little bit of problems	193 (49.0%)	63 (16.9%)	33 (50.0%)	22 (50.0%)	58 (48.3%)	34 (28.3%)	102 (49.0%)	7 (3.4%)			
Some problems	101 (25.6%)	6 (1.6%)	25 (37.9%)	0	37 (30.8%)	6 (5.0%)	39 (18.8%)	0			
A lot of problems	33 (8.4%)	2 (0.5%)	0	0	24 (20.0%)	2 (1.7%)	9 (4.3%)	0			
Extreme problems	0	0	0	0	0	0	0	0			

[a]Calculated using the chi-square test.

[b]The comparison of the responses of the guardians on EQ-5D-Y dimensions at admission and discharge.

[c]Children younger than 18 months were assumed to have no problem on walking about dimension.

Confidential: For Review Only
